# A multimodal human protein embeddings database: DeepDrug Protein Embeddings Bank (DPEB)

**DOI:** 10.1093/nargab/lqag042

**Published:** 2026-04-28

**Authors:** Md Saiful Islam Sajol, Magesh Rajasekaran, Hayden Gemeinhardt, Adam Bess, Chris Alvin, Supratik Mukhopadhyay

**Affiliations:** Department of Computer Science, Louisiana State University, Baton Rouge, LA 70803, United States; Department of Environmental Sciences and the Center for Computation and Technology, Louisiana State University, Baton Rouge, LA 70803, United States; Department of Computer Science, Louisiana State University, Baton Rouge, LA 70803, United States; Department of Computer Science, Louisiana State University, Baton Rouge, LA 70803, United States; Department of Computer Science, Furman University, Greenville, SC 29613, United States; Department of Environmental Sciences and the Center for Computation and Technology, Louisiana State University, Baton Rouge, LA 70803, United States

## Abstract

Computationally predicting protein–protein interactions (PPIs) is challenging due to the lack of integrated, multimodal protein representations. DeepDrug Protein Embeddings Bank (DPEB) is a curated collection of 22 043 human proteins that integrates four embedding types: structural (AlphaFold2), transformer-based sequence (BioEmbeddings), contextual amino acid patterns (ESM-2: Evolutionary Scale Modeling), and sequence-based n-gram statistics (ProtVec). AlphaFold2 protein structures are available through public databases (e.g. AlphaFold2 Protein Structure Database), but the internal neural network embeddings are not. DPEB addresses this gap by providing AlphaFold2-derived embeddings for computational modeling. Our benchmark evaluations show GraphSAGE with BioEmbedding achieved the highest PPI prediction performance (87.37% AUROC, 79.16% accuracy). The framework also achieved 77.42% accuracy for enzyme classification and 86.04% accuracy for protein family classification. DPEB supports multiple graph neural network methods for PPI prediction, enabling applications in systems biology, drug target identification, pathway analysis, and disease mechanism studies.

## Graphical Abstract

**Graphical Abstract:**
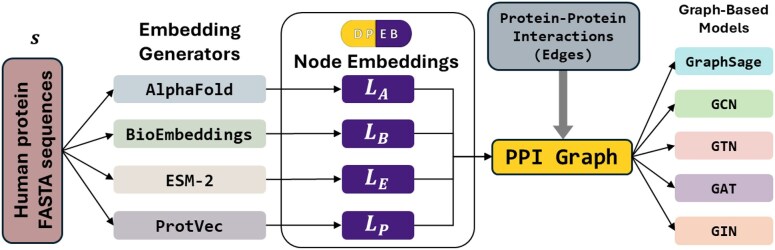

## Introduction

Protein–protein interactions (PPIs) are essential for cellular functions, including complex protein formation and cellular organization. The disruption of PPIs underlies numerous human diseases.

Experimental methods for studying PPIs are costly and slow when testing large numbers of protein pairs. With protein data from multiple databases, *in vivo* analysis of all potential PPIs is impractical. Computational methods fill that gap by providing a cost-effective alternative that complements experimental approaches.

### PPI databases

The human protein reference database (HPRD) [1] contains ~41 327 human PPIs that have been manually curated from published literature. In addition, it provides rich annotations detailing post-translational modifications, subcellular localization, and domain architecture.

BioGRID [2] offers a broader coverage, having grown to over 1 million human PPIs since its initial release. The Database of Interacting Proteins (DIP) [3] documents experimentally determined interactions, with 9141 human interactions. DIP includes a distinguished “core” subset for researchers requiring higher confidence data.

Context-specific interaction resources have also emerged. The Integrated Interactions Database [4] provides PPIs for 18 species across 133 tissues and 91 disease conditions, containing ~975 877 human PPIs (334 315 experimental and 667 804 predicted). HIPPIE [5] offers 273 900 experimentally detected interactions among 17 000 human proteins with filtering capabilities for tissue, function, and disease context.

BioPlex [6, 7] employs affinity purification-mass spectrometry to map the human interactome with 147 985 interactions among 12 695 proteins. APID [8] integrates PPIs from multiple primary databases with confidence scoring for over 400 000 annotated interactions. Despite their diversity and comprehensiveness, these specialized databases lack precomputed protein embeddings for computational modeling. Table 1 compares databases that provide protein embeddings alongside interaction data.

**Table 1.** Comparison of protein embedding and PPI databases

Dataset	Primary data type	Data modality	Graph/interaction data	Intended use cases	Species	Coverage info
DPEB	Multimodal embeddings: AlphaFold2, BioEmbeddings, ESM-2, ProtVec	Multimodal embeddings (latent space vectors)	Yes (11M PPIs from multiple sources)	Function prediction, PPI, graph-based architectures	Human-only (~22.4k proteins)	Human proteome (large-scale, up to 1975 FASTA sequences)
PCMol	AlphaFold2 latent embeddings for ~4331 proteins	Structural embeddings (latent space vectors)	No	*De novo* drug design, molecular generation	Multi-species (~4.3k)	4331 proteins (medium-sized, ligand data-rich subset)
AlphaFold2 DB	Predicted 3D atomic structures (PDB/mmCIF files)	3D structural coordinates and error estimates	No	Structural biology, visualization, modeling	Multispecies (incl. human)	>200M proteins from 47+ organisms
STRING	Network-based protein embeddings from STRING PPI	Network topology embeddings (interaction-based)	Yes (predicted associations)	Cross-species function prediction, network analysis	Multispecies (12k+ organisms)	1322 eukaryotes, thousands of proteins/species
BioPlex 3.0	Experimental PPI interactions (AP-MS data)	Physical protein–protein interaction networks	Yes (experimental PPIs)	Interaction mapping, complex identification	Human (HEK293, HCT116 cells)	118 162 interactions among 14 586 proteins, includes data from two human cell lines:

DPEB uniquely integrates four embedding types (AlphaFold2, BioEmbeddings, ESM-2, ProtVec) with 11M human PPIs, while other databases provide single modalities or lack embedding-interaction integration.

### Computational PPI prediction methods

Early approaches applied traditional machine learning techniques to custom protein representations. For instance, [9] implemented *k*-nearest neighbors with optimized distance measures on protein sequence descriptors, achieving 85.15% accuracy on yeast proteins but lacking evaluation on human proteins. Several works [10–13] employed support vector machines, reaching accuracies of 88%–91% on yeast proteins. Ensemble methods also showed promise, with [14] utilizing rotation forest classifiers and [15] implementing deep forest approaches for PPI classification. However, these approaches required manual feature engineering.

Deep learning methods automated feature extraction for PPI prediction. DNN-PPI [16] leverages deep neural networks to automatically learn protein representations from sequence-based descriptors, achieving an accuracy exceeding 94%. TAGPPI [17] combines TextCNN with Graph Attention Networks to extract and fuse both sequence and structural features. PIPR [18] uses a Siamese residual RCNN approach to extract local features and contextual information.

Graph neural networks (GNNs) reformulate PPI prediction as a link prediction task. GNN-PPI [19] captures topological patterns for previously unseen interactions. DL-PPI [20] combines Inception modules with attention mechanisms and a feature relational reasoning network. GNNGL-PPI [21] uses global and local graph features with graph isomorphism networks and protein embeddings derived from the MASSA model [22].

Recently, PCMol [23] demonstrated the utility of AlphaFold2-derived structural embeddings by conditioning molecular generative models on them for *de novo* drug design. They released a dataset of ~4300 protein embeddings of different species, limited to sequences up to 1536 amino acids. However, existing approaches rely on single-modality protein representations, lack human proteome specificity, or cannot integrate complementary embeddings.

### Our contribution

We present DeepDrug Protein Embeddings Bank (DPEB), which integrates four embedding types for 22 043 human proteins: AlphaFold2 [24] structural features, BioEmbeddings [25] sequence representations, ESM-2 [26] contextual patterns, and ProtVec [27] n-gram statistics. These complementary embeddings capture different aspects of protein structure and function. The multimodal approach provides more comprehensive protein characterization than single embedding methods.

DPEB enables comparison of predictions across multiple embedding types. Thus, researchers can identify consensus predictions or method-specific interactions. Different embedding types showed varying predictive accuracy, indicating each captures different protein properties. BioEmbedding achieved the highest AUROC (87.37%) in our experiments, while AlphaFold2 captured structurally driven interactions. No single embedding type captured all protein properties, demonstrating the value of multimodal approaches.

Table 1 shows that DPEB combines human-specific data from UniProt [28], STRING [29], and IntAct [30] with multimodal embeddings. DPEB supports multiple GNN architectures including GraphSAGE, GCNs, GTNs, GATs, and GINs. This enables researchers to select appropriate methods for specific biological questions.

## Materials and methods

**Figure 1.**
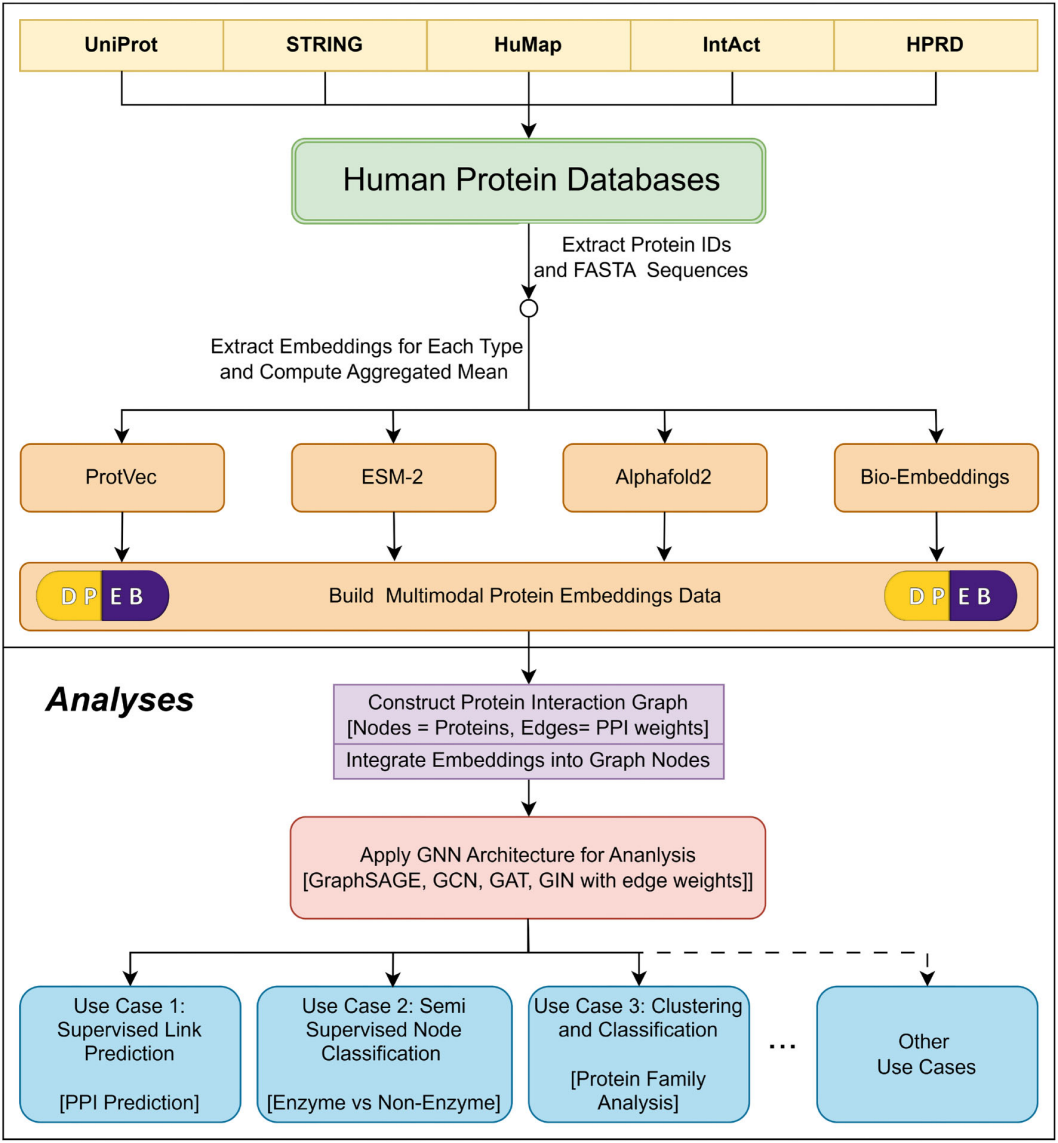
Overview of the DPEB pipeline for multimodal protein embedding analysis. Protein IDs and FASTA sequences are extracted from major human protein databases (UniProt, STRING, HuMap, IntAct, HPRD). Embeddings from four diverse sources—ProtVec, ESM-2, AlphaFold2, and BioEmbeddings—are computed and aggregated to build the DPEB (Deep Protein Embedding Benchmark) multimodal dataset. These embeddings can then be integrated into nodes of a PPI graph, where the edges denote interaction strengths represented by weighted connections. GNNs, including GraphSAGE, GCN, GTN, GAT, and GIN with edge weight support, are applied to perform downstream tasks such as supervised link prediction (PPI), semi-supervised node classification (e.g. enzyme versus non-enzyme), and protein family clustering and classification.

As shown in Fig. 1, DPEB can construct PPI graphs where users can select different protein embeddings and incorporate interaction data from external sources such as HuMap. Each protein functions as a node, with neighborhoods defined by interaction relationships. Nodes carry features derived from amino acid sequences and structural properties, while edges encode residue interaction information from PPI databases.

### Data sources and integration

Our framework integrates protein databases for sequence and interaction data.

#### Primary protein sequence data

The Universal Protein Resource (UniProt) [28] serves as our primary source for protein sequence data and functional annotations. UniProt provides curated annotations on protein structure, biological functions, and post-translational modifications. We leverage UniProt’s manually curated Swiss-Prot entries to ensure reliability in protein feature extraction. The database’s standardized nomenclature and cross-references enable integration with other datasets.

#### Protein–protein interaction data sources

The HPRD [1] includes experimentally validated interactions between protein pairs, curated from peer-reviewed studies. In addition to interaction data, it provides detailed biological annotations, including protein modifications, domain structures, and subcellular localization. HPRD contributes a high-quality subset of human interactions that complement large-scale high-throughput datasets and prediction-based repositories. Incorporating HPRD introduces manually reviewed interactions from published literature.

STRING (Search Tool for the Retrieval of Interacting Genes/Proteins) [29] is a widely used database that integrates both experimentally verified and computationally inferred PPIs. It aggregates data from diverse sources, including large-scale experimental data, predictive algorithms, text mining of scientific literature, and manually curated pathway databases.

A distinctive feature of STRING is its assignment of confidence scores to protein associations, representing the estimated probability that the supporting evidence corroborates the biological relevance of an interaction. It encompasses both direct molecular contacts and functional associations, providing broader context than databases focused solely on experimental binding evidence. With coverage of over 14 000 organisms, STRING offers extensive taxonomic breadth while maintaining comprehensive documentation of human–human protein interactions. This database provides annotations of both direct experimental evidence and interactions inferred from evolutionary conservation.

IntAct [30] is a freely accessible, open-source database that offers both molecular interaction data and integrated analysis tools. Unlike STRING’s scoring approach, IntAct focuses on manually curated, experimentally verified molecular interactions from literature or direct data submissions. The curation model employed by IntAct prioritizes comprehensive annotation of interaction experiments, including the specific methodologies used, experimental conditions, and quantitative interaction parameters. IntAct employs rigorous curation standards following International Molecular Exchange consortium guidelines, ensuring high data quality with comprehensive evidence trails. The database supports complex interaction networks beyond simple binary interactions, representing molecular complexes and their hierarchical organization.

HuMap [31] represents a specialized, high-confidence human protein interactome map that addresses the quality limitations inherent in many PPI datasets. HuMap integrates interactions detected by multiple orthogonal assays, substantially reducing false positives while maintaining extensive proteome coverage. In contrast to general PPI databases, HuMap is dedicated solely to human binary interactions, which are validated using complementary experimental techniques. The dataset employs standardized reliability scoring derived from experimental reproducibility metrics, allowing quality-based filtering for applications requiring different confidence thresholds. We use HuMap as a source of accurate ground truth data for protein interactions.

### Protein embedding methodologies

DPEB provides four distinct protein embedding strategies: structural, sequence-based, contextual, and statistical. Users can select different embedding types based on their particular analysis requirements.

#### Structural embeddings

AlphaFold2 [24] uses deep learning methods to predict protein structures. It integrates multiple sequence alignments (MSAs), evolutionary information, and attention-based neural architectures to address the protein folding problem. The system achieved accuracy comparable to experimental methods for many proteins at the 14th Critical Assessment of Protein Structure Prediction (CASP14). AlphaFold2 generates structural embeddings that capture spatial relationships among amino acids in protein conformations. These embeddings encode information about folding patterns, domain interactions, and potential binding sites. AlphaFold2 translates sequence information into structural predictions that maintain physical and biochemical plausibility.

To obtain structurally informed embeddings, we employed a customized implementation of the AlphaFold2 pipeline configured to operate in single-sequence mode. Specifically, we used the first model from the AlphaFold2 ensemble—Model 1 PTM, an optimized CASP14 monomer model that includes the predicted TM-score, detailed in Sections 1.11.1 and 1.12.1 of the Supplementary Information included with the original AlphaFold2 paper [24]. This model includes an additional head to predict the template modeling score (pTM), providing an estimate of global structural accuracy. As one of the five official AlphaFold2 configurations used in the CASP14 competition, it serves as a robust monomeric structure predictor. The model leverages template information and supports up to 5120 additional MSA sequences.

For each input protein sequence, AlphaFold2 was run in single-sequence mode using only the first pre-trained model (model_1_ptm) from its ensemble of five. This configuration bypasses the construction of MSAs, allowing the model to efficiently extract internal structural representations directly from individual sequences. From the model output, we extracted intermediate residue-level embeddings known as the single representation, a learned tensor that captures the contextual and structural characteristics of each amino acid position. These per-residue vectors were aggregated and stored as fixed-length protein embeddings. The resulting embeddings contain both structural and contextual (semantic) features, making them highly suitable for a variety of downstream computational tasks.

#### Sequence-based embeddings

ProtVec [27] uses natural language processing techniques to transform protein sequences into continuous vector representations. ProtVec conceptualizes proteins as sentences and amino acid *n*-grams as words, applying the skip-gram neural network model to learn relationships between local subsequences. Unlike traditional methods that use predefined biochemical properties or structural information, ProtVec learns (unsupervised) representations directly from protein databases.

ProtVec created vector spaces where similar protein motifs cluster together and vector arithmetic reveals biochemical relationships. Inspired by word2vec [32], this approach transforms variable-length protein sequences into fixed-dimensional vectors (typically 100 dimensions). These vectors are fed to machine learning models as input features for tasks such as protein classification, function prediction, and structural analysis. ProtVec applies distributional semantics to protein sequences, capturing protein biochemistry without explicit feature engineering.

In our implementation, we utilized a skip-gram model pre-trained on the Swiss-Prot database, allowing us to capture local sequence patterns commonly observed in biologically relevant proteins. For each protein, we computed embeddings using three offset tokenizations to account for different reading frames. The embeddings from each offset were averaged and then concatenated to produce a 300-dimensional vector per protein. This encoding strategy provides distributional representations by capturing local semantic patterns within overlapping *k*-mers. We computed ProtVec embeddings for all proteins in the dataset using this approach.

#### Language model embeddings

ESM-2 [26] embeddings are derived from a transformer-based model that is trained on billions of protein sequences using a self-supervised learning strategy. They capture evolutionary context without MSA and produce high-dimensional, residue-aware vectors suitable across diverse protein families. To derive protein-level representations from evolutionary-scale data, we employed the pre-trained transformer model esm2_t33_650M_UR50D from the ESM-2 family developed by Meta AI. Each protein sequence was formatted into standard FASTA and processed through the ESM-2 embedding pipeline. For every sequence, the model outputs token-level embeddings across 33 transformer layers. We used the final layer (layer 33) and applied mean pooling across all residue embeddings to obtain a fixed-length, 1280-dimensional vector for each protein. These embeddings capture both local and global contextual information, learned from large-scale corpora of protein sequences.

BioEmbeddings [25] provides a standardized pipeline generating protein embeddings from sequences. The framework implements various encoding strategies within a single infrastructure. We generate protein embeddings using a prottrans_bert_bfd LM model [33] trained on BFD large dataset [34]. This ProtTransBFD model uses BertTokenizer for tokenizing protein sequences.

BioEmbeddings generates embeddings at different granularities: per-protein embeddings for global characterization, per-residue embeddings for fine-grained analysis, and specialized embeddings for specific prediction tasks. The framework implements standardization procedures, ensuring vector representations maintain consistent dimensionality and numerical properties across different protein sequences without additional preprocessing. These embeddings encode both the order and context of amino acids in protein sequences.

#### Embedding dimensionality and normalization

In the DPEB repository, we share both the raw (per-residue) and aggregated (per-protein) versions of each embedding. The raw format retains the full contextual information captured by the models. For example, ESM-2 provides a 1280-dimensional vector for every amino acid. The aggregated version summarizes these using mean pooling to produce a fixed-length vector per protein (e.g. 1280-D for ESM-2, 384-D for AlphaFold2).

To preserve the integrity of the original models, we do not apply normalization or dimensional alignment across embedding types within the dataset. As a result, the dimensions vary: ProtVec (300-D), BioEmbedding (1024-D), ESM-2 (1280-D), and AlphaFold2 (384-D). We chose not to enforce uniformity of dimensionality because different tasks and models may benefit from different preprocessing strategies. Users can customize the data processing to their specific needs. This includes normalizing the embeddings, projecting them into a common space, or using each representation in its original form.

### Graph construction methodology

DPEB is constructed by unifying four major human PPI repositories: HPRD, HuMAP, IntAct, and STRING. Each dataset was preprocessed to retain only essential columns—namely the interacting protein pairs (source and target) and their associated interaction confidence scores (weight).

#### Data preprocessing and standardization

To ensure a consistent schema across datasets, column names were standardized and all entries were integrated into a single interaction list through concatenation. The resulting dataset comprises over 11 million interaction pairs, providing extensive coverage of the human interactome while reflecting varying confidence levels across different data sources.

#### Graph construction

A unified interaction graph was then constructed using the Deep Graph Library (DGL; https://www.dgl.ai/), where each unique protein was mapped to a numeric node index. Nodes in the graph were further enriched with high-dimensional protein embeddings derived from ProtVec, ESM-2, BioEmbedding, and AlphaFold2. These embeddings encode semantic and structural information for each protein node.

Edge weights were mapped directly from the confidence scores in the unified dataset, normalized to the [0, 1] range, and incorporated into the graph as a float32 tensor. These weights allow GNNs to consider the biological confidence associated of each PPI. Edges were filtered to include only those between proteins with valid embeddings. Duplicate interactions were removed to avoid data leakage during train-test splits. We saved the final graph and the protein-to-index mapping in Python pickle format for downstream analysis.

The detailed extraction processes and embedding pipelines are available in our public repository: DPEB.

#### Protein sequence characteristics

**Figure 2.**
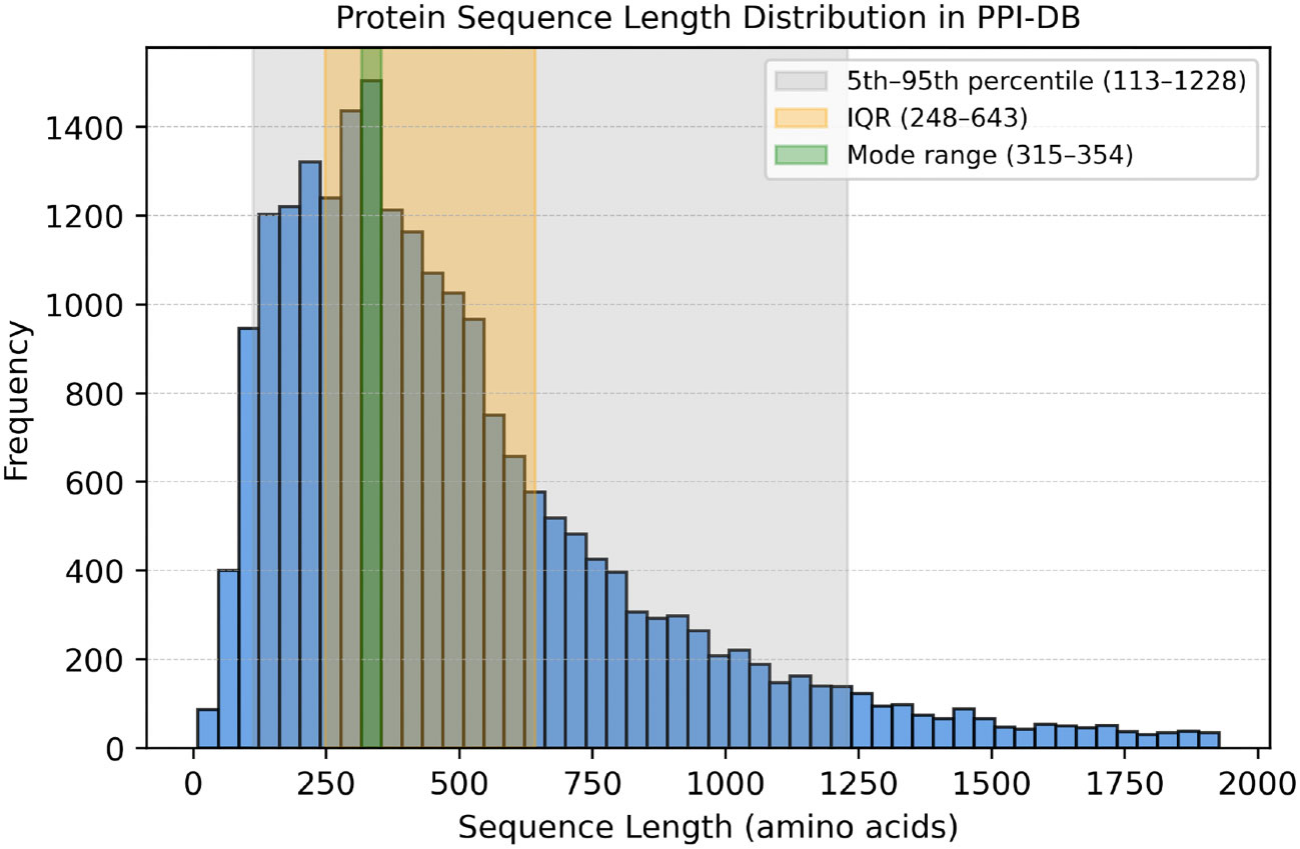
Distribution of human protein sequence lengths in the DPEB. Most sequences range from 113 to 1228 residues (5th–95th percentile), with the highest frequency observed between 315 and 354 residues.

As depicted in Fig. 2, the distribution of human protein sequence lengths in our dataset reveals characteristic patterns. The majority of sequences (90%) fall within the 113–1228 residue range (5th–95th percentile), with the most frequent lengths concentrated between 316 and 354 amino acids.

### Benchmark evaluation framework

DPEB supports multiple graph-based neural network architectures for benchmarking protein interaction methods.

#### Graph neural network architectures

We implemented and benchmarked five GNN architectures: GraphSAGE, GAT, GCN, GTN, and GIN. These architectures are commonly used for PPI prediction and biological network analysis [35–38].

We implemented the following architectures for protein interaction data:

- **GraphSAGE**: A scalable GNN that efficiently creates node embeddings through local neighborhood sampling and feature pooling.
- **Graph Attention Network (GAT)**: Uses attention mechanisms to weight neighbor contributions during node aggregation.
- **Graph Convolutional Network (GCN)**: Performs spectral convolution operations over nodes and their neighborhoods.
- **Graph Transformer Network (GTN)**: Combines self-attention mechanisms with graph structure to capture complex dependencies.
- **Graph Isomorphism Network (GIN)**: A powerful architecture designed to approximate graph isomorphism tests, enhancing the discriminative power of GNNs.

These architectures capture distinct aspects of protein interaction networks, from localized patterns to broader global graph structures.

#### Model evaluation approach

These baselines enable comparative analysis of different methods on human protein interaction prediction. We provide three example applications using different protein embeddings from DPEB: PPI prediction, functional classification, and structural analysis.

## Results

DPEB allows users to query protein embeddings using UniProt identifiers. Users can select from the four embedding types: AlphaFold2, BioEmbeddings, ESM-2, and ProtVec. The following subsections detail our three use cases.

### Use Case 1: Supervised protein–protein interaction link prediction

We predict PPIs using supervised link prediction problem on a human protein interaction graph with over 11 million known interaction pairs. We evaluate the performance of multiple GNN architectures with different protein embeddings.

#### Experimental setup

We treat protein interaction prediction as binary edge classification. The nodes depict proteins, while the edges represent known interactions with associated weights. For each positive interaction, we generate one negative sample by randomly selecting a protein pair without observed interactions [18, 20, 21, 39, 40, 41]. We exclude self-connections and maintain a 1:1 positive-to-negative ratio for balanced training.

We trained five GNN architectures—GraphSAGE, GAT, GCN, GTN, and GIN—to assess their effectiveness across different protein embedding modalities. All models use DGL’s NeighborSampler for mini-batch training and binary cross-entropy loss for edge prediction. Node features incorporate all four embeddings types from DPEB.

The dataset was partitioned into training (70%), validation (20%), and testing (10%) subsets using edge-level splitting with bidirectional interactions preserved. We fixed the random seed to 42 for reproducibility. The graph’s large scale (millions of edges) made cross-validation infeasible, so we used a fixed train/validation/test split for all experiments. To address the lack of cross-validation, we provide bootstrap evaluations in the Supplementary Tables S1–S4. Besides statistical significance testing using McNemar’s test across different embedding types is also provided in the Supplementary Tables S5–S9. We trained all models for 20 epochs with batch size 512 utilizing the Adam optimizer on an NVIDIA Tesla V100-SXM2 (32GB) GPU using PyTorch and DGL. Training took 7–8 h per model.

We tested learning rates of $\mathit {lr} = 10^{-3},\ 10^{-4},\ \mathrm{and}\ 10^{-5}$ for all models and report the best results for each embedding type. All models employed three graph convolution layers (or transformer layers for GTN) followed by an MLP-based edge predictor. The predictor computed interaction probabilities using element-wise multiplication of source and destination node embeddings.

#### Performance analysis and biological significance

We evaluated the models using F1-score, AUROC, and accuracy. Each metric was computed on the held-out test set using threshold-optimized binary predictions derived from predicted logits. Table 2 summarizes the results for each model-embedding combination. GraphSAGE with BioEmbedding yielded the highest AUROC of 87.37%, indicating the strongest ability in distinguishing between interacting versus non-interacting protein pairs. This configuration also achieved the best F1-score (0.7896) and overall accuracy (79.16%), outperforming even structure-aware embeddings such as AlphaFold2.

**Table 2.** Baseline F1, AUROC, and accuracy scores for GraphSAGE, GCN, GTN, GAT, and GIN using different protein embedding approaches on human protein datasets

Models	Embeddings	F1	AUROC	Accuracy
GraphSAGE	ProtVec	0.7579	84.26	75.88
	ESM-2	0.7604	84.26	75.94
	BioEmbedding	0.7896	87.37	79.16
	AlphaFold2	0.7616	84.47	76.17
	**Bio + ESM-2**	**0.7898**	**87.51**	**79.29**
GAT	ProtVec	0.7348	80.64	72.89
	ESM-2	0.7313	80.35	72.52
	BioEmbedding	0.7503	83.71	75.43
	AlphaFold2	0.7401	81.35	73.44
	**AlphaFold2 + Bio + ESM-2**	**0.7566**	**83.88**	**75.56**
GCN	ProtVec	0.6419	69.60	63.15
	ESM-2	0.6620	70.56	64.07
	BioEmbedding	0.6489	71.00	64.43
	AlphaFold2	0.6515	69.51	63.11
	**Bio + ESM-2**	**0.6885**	**74.67**	**67.73**
GTN	ProtVec	0.6876	73.94	67.07
	ESM-2	0.6872	72.63	65.90
	BioEmbedding	0.7154	79.32	71.53
	AlphaFold2	0.6935	74.58	67.71
	**Bio + ESM-2 + ProtVec**	**0.7347**	**82.08**	**74.18**
GIN	ProtVec	0.6495	70.31	63.90
	ESM-2	0.6543	71.09	64.44
	BioEmbedding	0.6382	68.84	65.21
	AlphaFold2	0.6392	70.37	63.81
	**AlphaFold2 + Bio + ESM-2**	**0.6860**	**73.58**	**67.13**

Models incorporate embeddings derived from AlphaFold2, ProtVec, ESM-2, and Bio Embeddings. For each model, an additional row reports the best-performing embedding combination, selected based on AUROC, with F1-score and accuracy shown for completeness. Extended results covering all evaluated single embedding types can be found in Supplementary Tables S1–S4, and multi-embedding combinations are available in the Supplementary Tables S14–S18, with overall best-performing combinations summarized in Supplementary Tables S19 and S20. McNemar statistical significance test results are provided in Supplementary Tables S5–S9.

GAT also benefited from BioEmbedding, achieving an AUROC of 83.71%. GTN performed competitively with AUROC values of 79.32% (BioEmbedding) and 74.58% (AlphaFold2). GCN consistently showed the weakest performance across all embedding types, likely due to its limited ability to capture higher-order neighborhood dependencies.

GIN showed moderate performance with AUROC around 70% on most embeddings, slightly underperforming GTN and GraphSAGE. Despite incorporating edge weight, GIN demonstrated suboptimal performance in the PPI task, likely due to its lack of attention mechanisms or adaptive neighborhood sampling strategies. Across all models, BioEmbedding consistently provided the most informative features, outperforming both unsupervised sequence models (ProtVec, ESM-2) and structure-aware embeddings (AlphaFold2). In contrast to AlphaFold2’s structural focus, BioEmbedding captures sequence-derived functional properties such as evolutionary context, remote homology, and sequence-order dependencies. This makes BioEmbedding particularly effective for PPI prediction.

In addition to single-modality embeddings, we evaluated multimodal concatenation settings, as given in Table 2. For several GNN architectures, concatenated embeddings yielded consistent gains over individual modalities—e.g. GraphSAGE with BioEmbedding + ESM-2 achieved the highest AUROC (87.51%) and accuracy (79.29%), while GTN benefited from BioEmbedding + ESM-2 + ProtVec with an AUROC of 82.08%. While the optimal combination varied across architectures (e.g. GraphSAGE, GAT, GTN, and GCN), multimodal representations consistently outperformed their single-embedding counterparts in terms of AUROC, F1-score, and accuracy. A comprehensive breakdown of performance across all evaluated embedding combinations and models is provided in the Ablation Study in the Supplementary Tables S14–S20. Overall, these results suggest that combining complementary sequence- and context-aware embeddings can improve PPI link prediction beyond any single representation shown in Table 2.

The generated predictions can act as preliminary hypotheses for wet-lab validation, enabling a cost-effective and scalable strategy to investigate protein relationships. BioEmbedding-based models capture functionally meaningful signals, making them especially valuable for understanding how protein interactions in pathways, complexes, disease contexts, and drug discovery.

### Use Case 2: Node classification for enzyme function prediction via semi-supervised learning

We performed binary classification to distinguish enzymes from non-enzymes using different protein embeddings from DPEB. The workflow used two sequential stages: unsupervised representation learning with a GCN, followed by supervised evaluation with logistic regression.

#### Representation learning and classification methodology

##### Stage 1: Unsupervised node representation learning

We constructed a PPI graph with 21 435 human proteins. Each node used one of five embedding types: AlphaFold2, ProtVec, ESM-2, BioEmbedding, or a concatenated vector (combining all four). All embeddings were L2-normalized except ESM-2, which is already normalized. Proteins were labeled as enzyme or non-enzyme based on UniProt annotations. The dataset contained 16 675 non-enzyme proteins and 4660 enzyme proteins, creating a moderate class imbalance that was addressed using class-weighted logistic regression during evaluation. We trained a three-layer GCN using self-supervised contrastive learning with CosineEmbeddingLoss. Positive pairs were from PPIs on the graph; negative pairs were randomly sampled uniformly. The architecture included batch normalization, GELU activations, 30% dropout, and final projection to a 32-dimensional embedding space. We trained for 200 epochs utilizing the Adam optimizer with the learning rate being $10^{-2}$.

##### Stage 2: Supervised enzyme classification

After obtaining node embeddings from the pre-trained GCN, we trained a logistic regression classifier to predict enzymatic status. The labeled protein dataset was segregated into training and testing sets with 80% for training and 20% for testing. We applied class balancing and feature standardization for fair evaluation. This evaluation quantified how well each embedding type preserved functionally relevant information in the unsupervised setting.

#### Results and functional implications

Table 3 presents the classification results across all five embedding configurations. As a baseline, we trained the GCN with constant node features to simulate the absence of protein-specific information. This setup yielded an accuracy of 61.7%, highlighting the contribution of graph structure alone. All biological embeddings outperformed this baseline, indicating the importance of protein-level information in enzyme classification.

**Table 3.** Performance comparison of different protein embeddings on enzyme versus non-enzyme classification using unsupervised GCN-based node embeddings followed by logistic regression

Embedding	Accuracy	Precision	Recall	F1-score
*Baseline*	0.6174	0.7615	0.6174	0.6509
AlphaFold2	0.7331	0.7869	0.7331	0.7506
ProtVec	0.6996	0.7781	0.6996	0.7227
ESM-2	0.7362	0.7975	0.7362	0.7547
BioEmbedding	0.7742	0.8221	0.7742	0.7886
Concatenated	0.7635	0.8078	0.7635	0.7778

The baseline uses constant node features without biological embeddings.

Among individual embeddings, BioEmbedding achieved the highest classification performance with 77.42% accuracy, 82.21% precision, and 78.86% F1-score. ESM-2 and AlphaFold2 embeddings also performed well, while ProtVec showed slightly lower performance. The concatenated embeddings (combining AlphaFold2, ProtVec, ESM-2, and BioEmbedding) achieved strong performance across all metrics (F1 = 77.78%), indicating that multimodal representations capture complementary biological signals.

This task demonstrates the capacity of DPEB to facilitate semi-supervised protein function prediction, where unsupervised pre-training is followed by supervised evaluation on specific biological tasks. The results indicate that embeddings from structural, sequence-based, and contextual models preserve meaningful biochemical information relevant to enzyme function. The improvement observed with concatenated embeddings suggests that combining orthogonal views of protein data can enhance functional resolution. These findings support DPEB as a versatile and biologically informative resource for graph-based machine learning applications in proteomics.

### Use Case 3: Embedding-based protein family clustering and multi-class classification

To investigate the biological relevance and discriminative capacity of AlphaFold2-derived embeddings, we performed a dual analysis involving unsupervised clustering and supervised classification of protein families. We used proteins from the 17 most frequently occurring human families in our dataset, each containing at least 60 members (total 2399 proteins). The goal was to understand whether family-specific structure is encoded in the raw AlphaFold2 embeddings and whether this structure can be enhanced via deep learning-based embedding refinement.

#### Unsupervised clustering and task-specific embedding refinement

Unsupervised clustering of raw AlphaFold2 embeddings. We applied unsupervised dimensionality reduction to visualize the structure of the raw AlphaFold2 embedding space. We employed t-distributed Stochastic Neighbor Embedding (t-SNE) to map the high-dimensional representations into two-dimensional space. The K-means clustering divided the proteins into clusters, with the number of clusters being the same as the number of known families.

**Figure 3.**
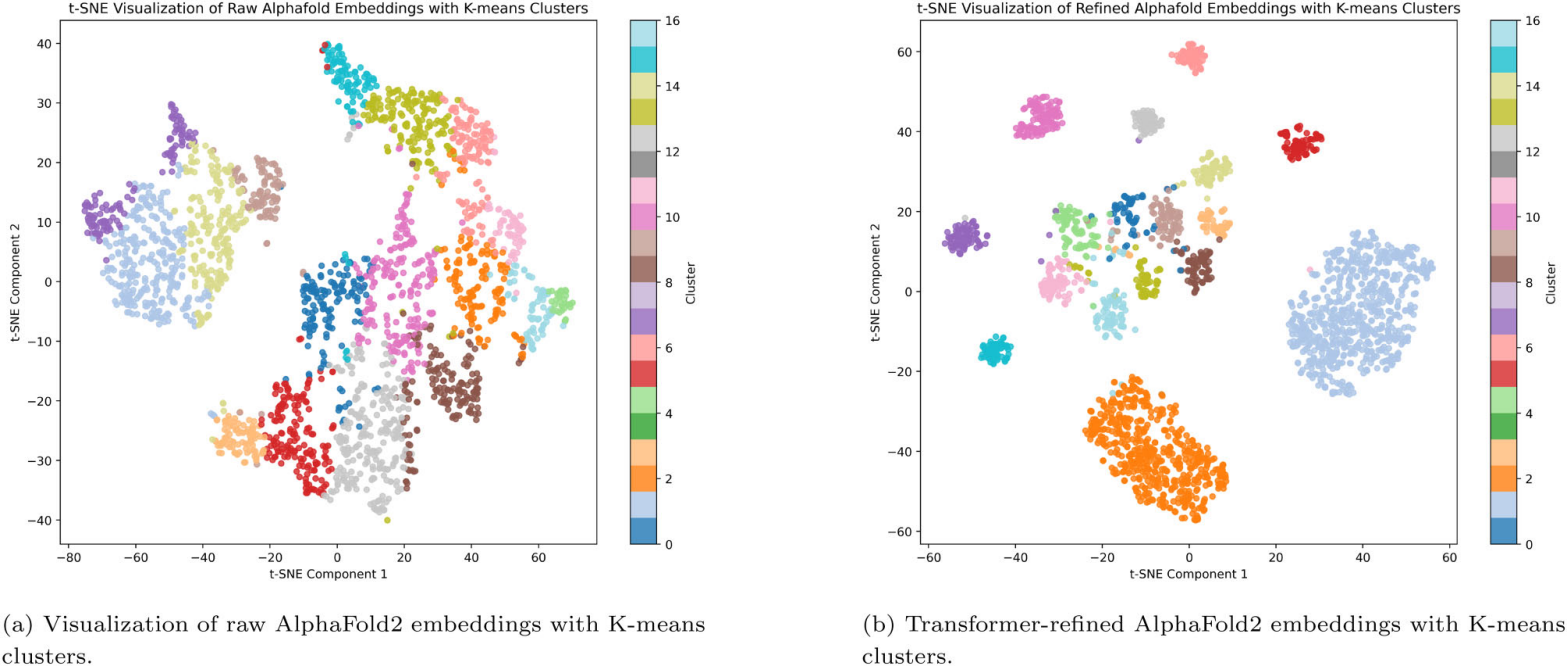
t-SNE comparison of protein embeddings with protein families distinguished by color. Each figure shows 2399 human proteins from the 17 families listed in Table 5. Additional t-SNE visualizations, including comparisons of raw versus refined embeddings across other modalities are provided in the Supplementary Fig. S1.

As visualized in Fig. 3a, some grouping is evident, but many clusters remain diffuse or overlap. This suggests that while raw AlphaFold2 embeddings carry some family-level information, the boundaries between families are not distinctly separated in the original space.

##### Task-aware refinement of AlphaFold2 embeddings using transformer encoder

To improve the family separability of the embedding space, we trained a transformer-based encoder that refines AlphaFold2 embeddings using protein family labels as supervision. This refinement process reduces noise and redundancy in raw protein representations and emphasizes discriminative features while suppressing less informative dimensions. This creates a more focused embedding space where evolutionary and functional relationships between protein families become clearer in the t-SNE visualization.

Our methodology employed a LayerNorm and a linear projection from 384 to 128 dimensions, followed by a two-layer transformer encoder (with 4 attention heads and 512-dimensional feedforward networks), and a classification head over 17 family classes. The network was trained using cross-entropy loss and the Adam optimizer (learning rate $10^{-4}$) for 600 epochs with an 80:20 train-test split and Xavier uniform weight initialization.

**Figure 4.**
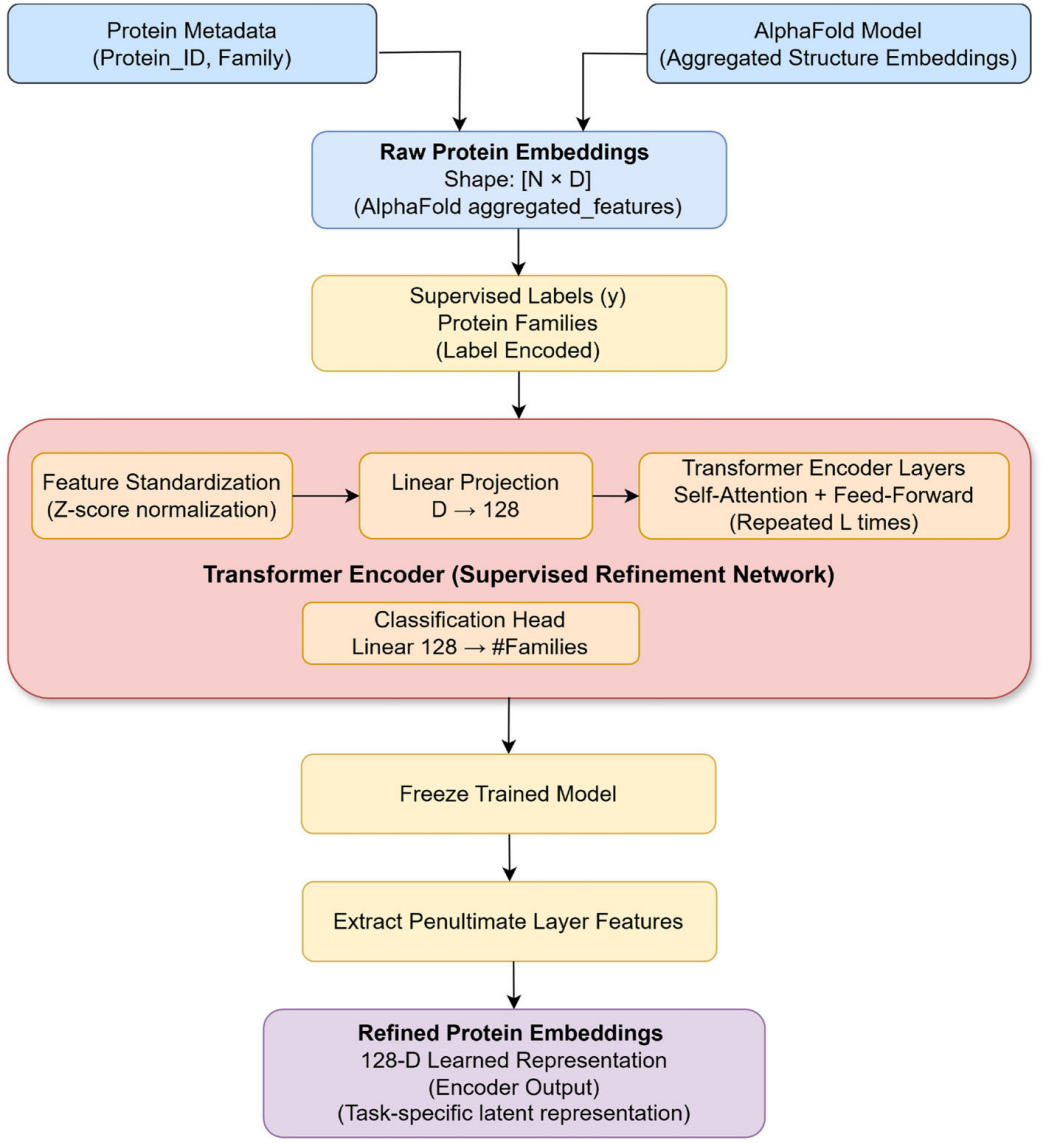
Flowchart illustrating the refinement of AlphaFold2 embeddings via supervised representation learning, where a neural network trained on protein family labels is used to extract task-aware latent embeddings from the penultimate layer.

The overall methodology for task-specific embedding refinement and evaluation is illustrated in Fig. 4, which presents the supervised transformer-based refinement pipeline used to convert raw AlphaFold2 embeddings into task-aware latent representations. As shown in the figure, protein family labels are used only during supervised training to guide the encoder toward family-discriminative features, while refined embeddings are extracted from the penultimate layer and reused for downstream analysis. This design ensures that refinement enhances biological separability without directly encoding label information into unsupervised analyses. After training converged, the learned transformer encoder was frozen and subsequently applied in inference mode to generate refined embeddings for the entire dataset, including both training and test proteins. Generating refined embeddings for the complete dataset is essential for evaluating the global structure of the learned embedding space. Since the objective of this analysis is to assess how well supervised refinement reshapes embedding geometry at the family level, restricting visualization or clustering to only a subset of proteins would provide an incomplete and potentially misleading representation of embedding separability. This procedure ensures that refined embeddings for all proteins are produced using a single, fixed representation function learned from the training data, without further parameter updates.

**Figure 5.**
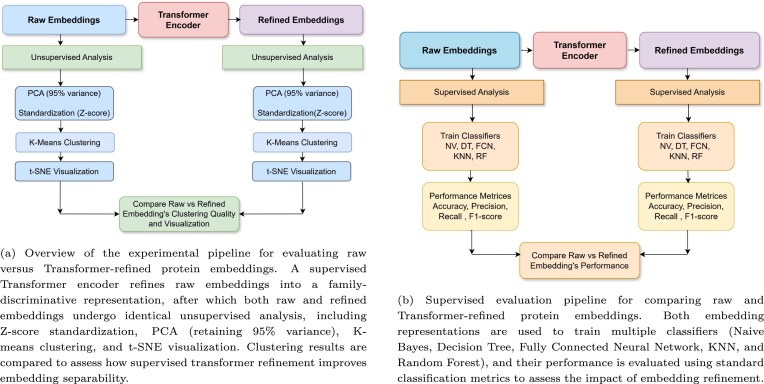
Overview of unsupervised and supervised evaluation pipelines used to compare raw and transformer-refined protein embeddings.

In addition, Fig. 5 provides a comprehensive overview of the experimental pipelines employed to compare raw and refined embeddings under both unsupervised and supervised settings. Specifically, Fig. 5a outlines the unsupervised evaluation workflow, where raw and refined embeddings undergo identical preprocessing steps—including Z-score normalization, Principal Component Analysis (PCA)-based dimensionality reduction, K-means clustering, and t-SNE visualization—allowing a fair assessment of embedding geometry and cluster separability. On the other hand, Fig. 5b demonstrates the supervised classification pipeline, in which multiple classifiers are trained on raw versus refined embeddings to quantify performance gains attributable to refinement. Together, these figures contextualize the visual and quantitative results presented in this section and clarify the strict separation between supervised training, unsupervised visualization, and downstream evaluation.

We extracted refined 128-dimensional embeddings from the output of the transformer encoder and re-evaluated their cluster structure using PCA, t-SNE, and K-means clustering. As shown in Fig. 3, the refined embeddings exhibit highly compact and well-separated clusters, showing significantly improved alignment with known family labels. The model follows the structure described in Table 4. This marks a substantial improvement over the raw embeddings, demonstrating that the transformer effectively learns to amplify family-discriminative features embedded in AlphaFold2 representations.

**Table 4.** Transformer encoder architecture used for refining 384-dimensional AlphaFold2 embeddings into 128-dimensional task-specific representations

Layer	Output dim	Description
LayerNorm	384	Layer normalization on input AlphaFold2 embeddings
Linear (Projection)	128	Fully connected layer; Xavier initialization
Transformer Encoder Layer 1	128	**Multi-head Self-Attention:** 4 heads, dimension of the key/query vectors, $d_k = 32$ per head **Feed-Forward Network:** Linear $\rightarrow$ 512 units $\rightarrow$ GELU $\rightarrow$ Linear $\rightarrow$ 128 units **Dropout:** 0.3 (applied after attention and FFN) **LayerNorm:** applied after each sub-layer
Transformer Encoder Layer 2	128	Same structure as Layer 1 (shared hyperparameters, no weight sharing)
Refined Embedding Output	128	Final output used for downstream clustering and classifier input
Linear (Classifier Head)	17	Linear layer mapping to class logits; softmax applied in loss function

The model consists of a normalization layer, a projection into a latent space, two self-attention layers with feed-forward networks, and a classification head. Final embeddings are extracted before the classifier.

We performed unsupervised K-means clustering on both the raw and refined embeddings. Prior to clustering, the high-dimensional embeddings were reduced using PCA to retain 95% of the variance, a standard practice to denoise and compress representations. K-means clustering was used with the same number of clusters as the number of ground-truth families. Clustering quality was evaluated using permutation-invariant agreement metrics, including Adjusted Rand Index (ARI), Normalized Mutual Information (NMI), and homogeneity–completeness–V-measure. These metrics quantify the degree of correspondence between cluster assignments and known protein family labels without requiring explicit cluster-to-label alignment and are therefore well-suited for clustering evaluation. Additionally, t-SNE plots were generated to visualize the clustering structure in two dimensions (Fig. 3), showing that refined embeddings yield more compact and separable clusters compared to their raw counterparts. The t-SNE projection is performed in a fully unsupervised manner, without access to family labels. Labels are used solely to color points in the 2-D visualization for interpretability and are not provided as input to t-SNE. Here, t-SNE is employed strictly as a post-hoc visualization tool and does not influence training, clustering, or quantitative evaluation; this procedure does not introduce label leakage. Quantitative clustering metrics are computed in the original high-dimensional embedding space, not on the t-SNE projection.

#### Comparative analysis and downstream classification performance

We considered families containing >60 members of human proteins, resulting in a subset of 2399 proteins, as shown in Table 5. As can be observed in Fig. 3, t-SNE visualization offered advantages through its preservation of local structure while allowing divergent relationships with greater distances in the reduced-dimensional space. Unlike PCA, which uses linear projections that often fail to capture non-linear relationships between protein families, t-SNE effectively revealed the distinct clustering patterns in our refined embeddings.

**Table 5.** Distribution of major human protein families analyzed, showing the 17 largest families with at least 60 members each, totaling 2399 proteins in the dataset

Protein family	Count
G-protein coupled receptor 1 family	681
Krueppel C2H2-type zinc-finger protein family	537
Peptidase S1 family	114
Tyr protein kinase family	95
Protein-tyrosine phosphatase family	93
Rab family	90
Intermediate filament family	90
CMGC Ser/Thr protein kinase family	81
CAMK Ser/Thr protein kinase family	78
Peptidase C19 family	76
DEAD box helicase family	75
Ser/Thr protein kinase family	73
Mitochondrial carrier (TC 2.A.29) family	68
Short-chain dehydrogenases/reductases (SDR)	63
STE Ser/Thr protein kinase family	63
TRIM/RBCC family	62
AGC Ser/Thr protein kinase family	60
Total	2399

When comparing Fig. 3(a) versus (b), the refined embeddings showed marked improvement in cluster definition with clearly separated and concentrated data points. The transformer effectively emphasized signals that distinguish protein families while minimizing less relevant variations. This refinement created embeddings that formed dense clusters with clear boundaries, highlighting stronger intra-family similarities and accentuating inter-family differences. The density patterns in the refined embedding visualizations provide potential insights into hierarchical relationships between protein families, with proximal clusters often representing evolutionarily or functionally related families.

##### Supervised protein family classification on raw versus refined embeddings

As shown in Fig. 5b, we implemented a supervised multi-class classification pipeline to evaluate the discriminative power of protein embeddings for predicting biological family. We tested well different types of protein embeddings—both raw and refined—can classify proteins into their correct functional families.

We used a high-quality subset of proteins, filtered to include only families with >60 members to ensure balanced representation across classes. Target labels were derived from curated UniProt annotations, and we applied label encoding to convert string-based family names into numeric class indices for classification.

To benchmark performance, we evaluated classifiers spanning classical machine learning and deep learning approaches. Each classifier was trained and tested on two embedding types: (i) raw high-dimensional AlphaFold2 embeddings and (ii) refined embeddings from a transformer encoder trained to enhance class separability. We standardized all embedding vectors using StandardScaler and used a fixed random seed for 80/20 splits. Classical classifiers used scikit-learn’s implementations, while neural models used PyTorch with Adam optimizer, dropout, and learning rate scheduling. The fully connected network (FCN) used five dense layers with GELU activations and batch normalization for stable training. In contrast, the transformer used two self-attention layers with a 128-dimensional projection layer. We evaluated models on the held-out test set using accuracy, precision, recall, and F1-score with weighted averaging to address class imbalance. Table 6 shows consistent improvement using refined embeddings. The highest performance came from transformer-refined embeddings combined with FCN and KNN.

**Table 6.** Comparison of classification metrics using raw versus refined AlphaFold2 embeddings across multiple models

	Raw embeddings				Refined embeddings			
Model	Accuracy	Precision	Recall	F1-score	Accuracy	Precision	Recall	F1-score
Naive Bayes	0.7562	0.7763	0.7562	0.7591	0.8521	0.8751	0.8521	0.8605
Decision tree	0.6979	0.7390	0.6979	0.7109	0.7583	0.7760	0.7583	0.7567
FCN	0.7688	0.7693	0.7688	0.7517	0.8729	0.8764	0.8729	0.8717
KNN	0.8000	0.8216	0.8000	0.8048	0.8833	0.8883	0.8833	0.8833
Random forest	0.8438	0.8422	0.8438	0.8393	0.8771	0.8852	0.8771	0.8779

Refined embeddings consistently improve F1-score and accuracy across most classifiers. Detailed K-means clustering performance results with ARI/NMI scores are given in the Supplementary Table S10. In addition, Supplementary Tables S11–S13 report classification performance comparisons of raw versus refined BioEmbeddings, ESM-2, and ProtVec representations across multiple classifiers, demonstrating that the refinement strategy generalizes beyond AlphaFold2.

Overall, this analysis demonstrates that task-specific supervised transformer refinement substantially enhances family-level structure in the embedding space. Importantly, our methodology maintains strict separation between supervised training, unsupervised visualization, and quantitative evaluation.

##### Embedding refinement enhances biological discrimination

Raw AlphaFold2 embeddings, despite being powerful structural representations, are unsupervised and task-agnostic [24]. They encode broad structural features without optimizing for functional or evolutionary distinctions such as protein family classification. Although these representations retain biologically relevant information, their discriminative capacity is reduced by task-irrelevant variance. Embedding refinement addresses this by introducing supervised signals that guide the latent space toward biologically meaningful organization. Refined embeddings provide more functionally discriminative representations of proteins, bridging the gap between structure-derived features and functional annotation tasks.

## Discussion

Understanding and predicting PPIs remains a major challenge in computational biology [42, 43] and drug discovery [44–48], largely because molecular relationships are complex and multifaceted. We introduced DPEB, a human-specific, multimodal PPI resource that integrates diverse embeddings—AlphaFold2, ProtVec, ESM-2, and BioEmbedding—within a unified graph framework. Table 7 provides a detailed comparison across three use cases, summarizing their task objectives, methods, embedding types, and key outcomes.

**Table 7.** Comparison of three benchmark use cases supported by DPEB: task objectives, methods, models, embeddings, and key findings

Use case	Task	Approach	Embeddings used	Key findings
**Use Case 1: Supervised PPI link prediction**	Predict whether a pair of proteins interact	Supervised edge classification using GNNs with binary cross-entropy loss	AlphaFold2, ProtVec, ESM-2, BioEmbedding	BioEmbedding with GraphSAGE achieved highest AUROC (87.37%), F1-score (0.7896), and accuracy (79.16%). Structure-based embeddings like AlphaFold2 were less effective.
**Use Case 2: Semi-supervised enzyme classification**	Classify proteins as enzymes or non-enzymes	Self-supervised GCN to learn node embeddings followed by logistic regression	AlphaFold2, ProtVec, ESM-2, BioEmbedding, concatenated	BioEmbedding achieved highest accuracy (77.42%). Concatenated embeddings also performed well, proving that different embeddings complement each other.
**Use Case 3: Protein family clustering and classification**	Task specific protein embeddings refinement and downstream task on protein functional family classification	Transformer-based embedding refinement followed by t-SNE+KMeans clustering and multi-class classification	AlphaFold2 (raw and refined) and all other	Refined AlphaFold2 embeddings showed clear cluster separation and improved classification (F1-score: 0.8833 with KNN, 87%+ with FCN/RF).

BioEmbeddings consistently performed best across tasks, particularly with GraphSAGE, likely because its transformer-based architecture captures contextual sequence patterns that correlate with protein function. AlphaFold2 provides structural insights, but its raw embeddings were less effective for link prediction until supervised refinement improved performance in protein family classification. Concatenated embeddings outperformed individual embeddings in enzyme classification, showing that combining biological features improves results. GraphSAGE and GTN outperformed GCN, demonstrating that complex models better capture interaction patterns. These findings establish DPEB as a powerful and adaptable platform for advancing PPI prediction and protein function analysis.

Across link prediction, enzyme classification, and family clustering, BioEmbeddings consistently provided the most informative features, while structural embeddings required refinement for optimal performance. This functional context proves essential for protein analysis. Approaches that integrate both structural and functional information consistently outperform methods relying on structure alone.

The performance differences between model architectures warrant discussion. The strong performance of GraphSAGE—achieving up to 87.37% AUROC in PPI prediction—demonstrates the efficacy of neighborhood sampling for capturing informative patterns within biological networks. GraphSAGE scales efficiently to large graphs while preserving local neighborhood structure, making it well-suited for protein interaction networks. Graph Transformer Networks also performed strongly, highlighting how attention mechanisms capture the hierarchical and diverse nature of protein relationships.

In contrast, GCN models showed relatively weaker performance (AUROC consistently below 71%), indicating limitations in modeling complex dependencies in protein interaction networks. Message passing limited to immediate neighborhoods fails to capture the multi-scale and hierarchical patterns in biological systems. Hybrid architectures that combine GraphSAGE’s scalable neighborhood sampling with attention mechanisms may further improve accuracy. Such approaches may enable more accurate and biologically grounded protein interaction analyses.

Protein family classification shows how refinement improves embeddings. Supervised fine-tuning increased classification accuracy to 86.04%, proving that task-specific training enhances structural embeddings for biological tasks. Iterative, task-specific refinement could improve protein embeddings for drug target prediction and pathway analysis.

A key contribution of this work is demonstrating how different embedding approaches complement each other. Multimodal representations outperformed single embeddings, particularly in enzyme classification. This indicates that individual embedding types cannot fully capture protein complexity. Future methods should integrate sequence, structural, and functional information for better protein representation.

Several limitations of DPEB should be acknowledged. Our focus on human proteins, while providing domain specificity, limits cross-species applicability. The static nature of pre-computed embeddings limits their ability to reflect context-dependent protein properties, particularly those that vary across different cellular states or environmental conditions. Dynamic embedding approaches that adjust representations based on biological context are an important direction for future research. In the PPI link prediction task, negative samples were generated by randomly selecting protein pairs without reported interactions. While this strategy is standard in large-scale PPI benchmarks [18, 20, 21, 39, 40, 41], unobserved interactions may include true positives, introducing label noise and potentially inflating absolute performance estimates. Nevertheless, since the same negative sampling protocol is applied consistently across all models and embedding types, the reported results remain reliable for relative performance comparison. Future research may explore more biologically informed negative sampling strategies, such as degree-aware sampling or confidence-weighted negatives, to further improve evaluation criteria.

DPEB makes several design decisions that differentiate it from existing protein databases. By combining embedding diversity with graph-based learning methods, we provide researchers with flexibility in modeling protein relationships. A key strength of DPEB lies in its embedding access framework (see the “Data availability” section), allowing researchers to retrieve different embeddings by UniProt IDs and enabling integration into downstream analyses. Beyond PPI prediction and functional annotation, DPEB can be extended to additional bioinformatics applications. In particular, the multimodal, human-specific protein embeddings provided by DPEB are well suited for cancer gene identification. Learned protein representations can be integrated with disease-specific interaction networks to prioritize oncogenes and tumor suppressors, as demonstrated in recent deep learning studies [49]. Similarly, DPEB offers a natural foundation for protein complex prediction, where embeddings can serve as node features in graph-based or transformer-based models to infer multi-protein assemblies and higher-order interaction patterns [50].

Future development of DPEB will focus on expanding coverage to include more human proteins, incorporating new embedding techniques as they emerge, and developing architectures that leverage multimodal protein representations more effectively. We plan to extend the framework to enable dynamic updating of protein embeddings as new structural and functional data becomes available.

DPEB serves as a resource for advancing PPI research. Its scalability, multimodal approach, and design enable applications in systems biology, drug discovery, and personalized medicine.

**Figure 6.**
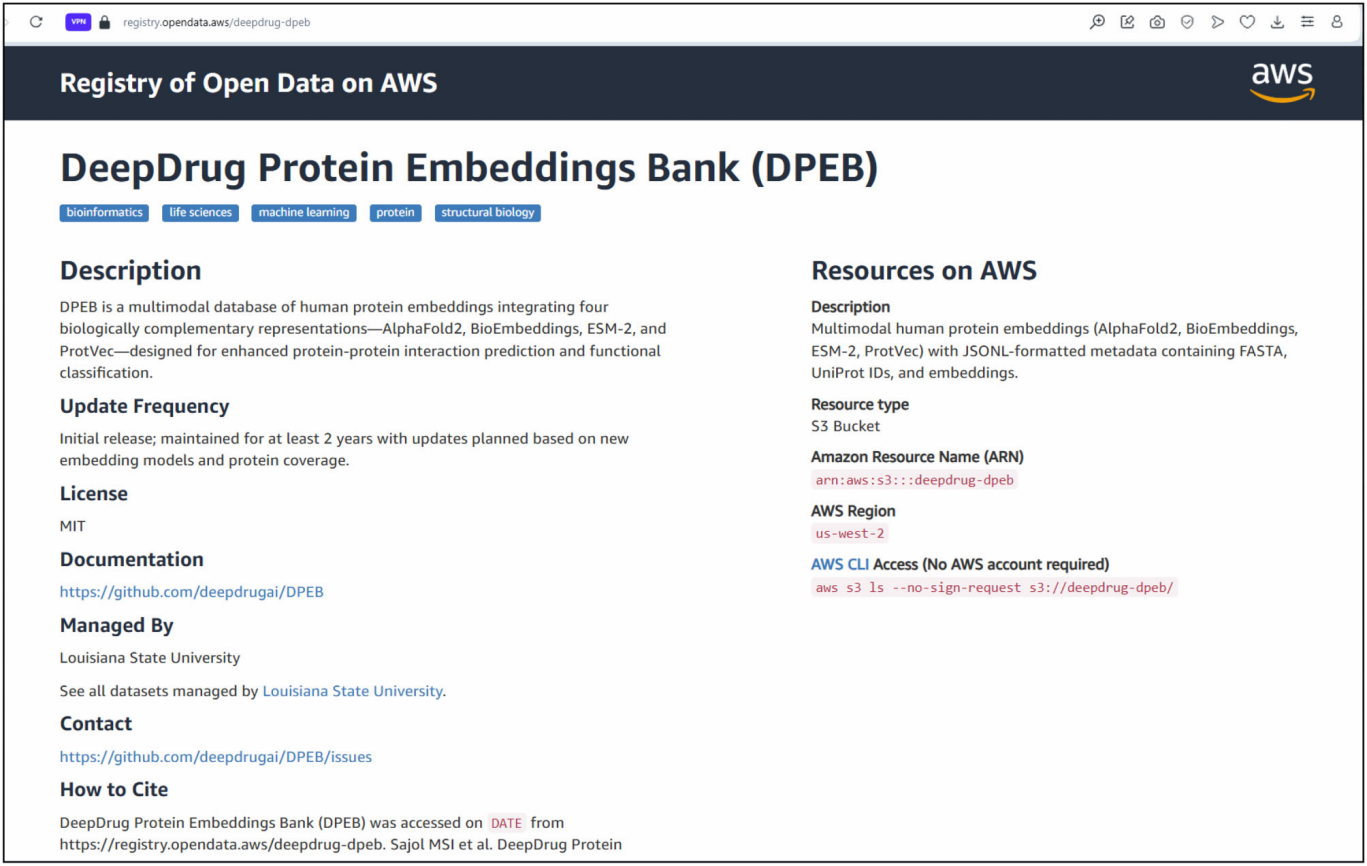
Snapshot of AWS Open Data page for the DPEB dataset. The resource provides multimodal human protein embeddings (AlphaFold2, BioEmbeddings, ESM-2, ProtVec) with associated metadata, hosted under the AWS Open Data Sponsorship Program. Available at https://registry.opendata.aws/deepdrug-dpeb/.

## Supplementary Material

lqag042_Supplemental_Files

## Data Availability

DPEB is publicly available through the AWS Open Data Program (https://registry.opendata.aws/deepdrug-dpeb/), which is an Amazon Web Services initiative that supports free, large-scale, and long-term hosting of open scientific datasets. Figure 6 provides a snapshot of the DPEB registry page used for dataset access and documentation. This public landing page provides access to the multimodal embedding files. Detailed usage instructions are given in the Dataset Accessibility section of the Supplementary Information. All embeddings provided in DPEB correspond to raw general-purpose representations generated from foundation models, including AlphaFold2, ESM-2, BioEmbedding, and ProtVec. Source code for task-specific refinement is also included. The database is designed to support diverse downstream applications such as PPI prediction, enzyme classification, and protein family analysis. The source code and scripts for reproducing the experiments in this paper are publicly available through Zenodo. The archived repository can be accessed at https://doi.org/10.5281/zenodo.19008090. The corresponding development version of the project is maintained on GitHub at https://github.com/deepdrugai/DPEB. The Zenodo archive provides a permanent, citable snapshot of the repository associated with this publication. The GitHub repository provides detailed documentation for loading and using the dataset, including instructions for downloading the data via the AWS CLI, example workflows using Google Colab, sample tutorials demonstrating typical usage, and updates regarding future development and extensions of the DPEB project. Additionally, detailed documentation of the dataset structure, accessibility, and programmatic access is provided in the Supplementary Information. The resource will be actively maintained with updates planned as new embedding models and expanded protein coverage become available. We designate the present release of the dataset as DPEB v1.0.1. All files corresponding to this release are permanently archived in the versioned directory s3://deepdrug-dpeb/v1.0.1/. Future updates will be deposited under incremented version labels (e.g. v1.0.2, v2.0.0), ensuring that past releases remain accessible for reproducibility of published analyses. The dataset is released under the Creative Commons Attribution 4.0 International License (CC-BY 4.0), permitting unrestricted use, distribution, and reproduction in any medium, provided the original work is properly cited. Source codes used for data processing and analysis is released under the MIT License (MIT), ensuring free and open use for both academic and industrial research.
