# Supplementary material for: A multimodal human protein embeddings database: DeepDrug Protein Embeddings Bank (DPEB)
Source: NAR Genom Bioinform. 2026 Apr 28;8(2):lqag042. doi: 10.1093/nargab/lqag042 (PMC13137052; doi:10.1093/nargab/lqag042)
Supplement: lqag042_Supplemental_Files [file lqag042_supplemental_files.zip › DPEB Supplementary.pdf]

*Nucleic Acids Research,* 2025, pp. 1–9

doi: DOI HERE
Advance Access Publication Date: Day Month Year
Paper

PAPER

# Supplementary Information: A Multimodal Human Protein Embeddings Database: DeepDrug Protein Embeddings Bank (DPEB)

Md Saiful Islam Sajol,[1] Magesh Rajasekaran,[3] Hayden Gemeinhardt,[1] Adam Bess,[1] Chris Alvin[2,*] and Supratik Mukhopadhyay[3]

[1]Department of Computer Science, Louisiana State University, Baton Rouge, LA, 70803, USA, [2]Department of Computer Science, Furman University, Greenville, SC, 29613, USA and [3]Department of Environmental Sciences and the Center for Computation and Technology, Louisiana State University, Baton Rouge, LA, 70803, USA

[*]Corresponding author. chris.alvin@furman.edu

FOR PUBLISHER ONLY Received on Date Month Year; revised on Date Month Year; accepted on Date Month Year

## Detailed Results of Use Case 1: Supervised Protein-Protein Interaction Link Prediction

### Robustness Analysis of Graph Neural Network Models

Supplementary Tables 1 through 4 report the robustness analysis of five graph neural network (GNN) models—SAGE, GAT, GCN, GTN, and GIN—evaluated across four distinct protein embedding types: AlphaFold2, BioEmbedding, ESM-2, and ProtVec. Each supplementary table corresponds to one model and presents the mean performance scores obtained from 100 independent bootstrap evaluations, along with their respective standard deviations and 95% confidence intervals.

This analysis aims to assess the average predictive performance, variability, and reliability of each model when subjected to random perturbations in the test edge set. Rather than relying on a single static test split, this bootstrap-based strategy provides a more comprehensive understanding of how sensitive or robust each model-embedding combination is under edge re-sampling, which more closely reflects deployment scenarios involving incomplete or noisy interaction data.

To generate these results, we used a bootstrapped evaluation pipeline in which each model was evaluated over 100 randomized test sets, each created by sampling with replacement from the original held-out edge list. For each bootstrap iteration, the model inferred interaction scores between protein pairs, which were then thresholded into binary predictions using Youden's J statistic derived from the ROC curve. Standard classification metrics—including Accuracy, Area Under the Curve (AUC), Precision, Recall, and F1-score—were computed for each run. The final values reported in Supplementary Tables 1–4 reflect the average performance over these 100 iterations, along with the empirical standard deviation and the 2.5th to 97.5th percentile confidence intervals.

Across all models, AlphaFold2 embeddings yielded notably stable and competitive performance. In particular, GAT and GCN models achieved high AUC and F1-scores when using AlphaFold2 features, often on par with or exceeding those obtained from ESM-2 and BioEmbedding. Furthermore, the standard deviations of metrics for AlphaFold2 embeddings were consistently low, highlighting their robustness under repeated test perturbations. This suggests that AlphaFold2-derived structural embeddings provide not only biologically rich features but also enhance the consistency of link prediction models under noisy or incomplete edge scenarios.

In contrast, while ProtVec exhibited reasonable performance in some configurations, its variability was higher and its average metrics were lower compared to AlphaFold2 and ESM-2, especially for the GTN model, which showed sensitivity to embedding quality. ESM-2 and BioEmbedding remained strong overall, with BioEmbedding showing particularly high precision in the GAT-based evaluations.

**Supplementary Table 1.** Performance of GNN Models with **AlphaFold2** Embeddings.

| Model | LR | Acc | Acc Std | Acc CI | AUC | AUC Std | AUC CI | Prec | Prec Std | Prec CI | Recall | Recall Std | Recall CI | F1 | F1 Std | F1 CI |
|---|---|---|---|---|---|---|---|---|---|---|---|---|---|---|---|---|
| SAGE | 1e-3 | **76.12** | 0.0293 | [76.0737, 76.1834] | **0.8447** | 0.0003 | [0.8443, 0.8452] | **0.7624** | 0.0016 | [0.7588, 0.7657] | **0.7590** | 0.0031 | [0.7529, 0.7650] | **0.7607** | 0.0008 | [0.7592, 0.7622] |
| GAT | 1e-3 | 74.41 | 0.0310 | [74.3480, 74.4618] | 0.8246 | 0.0003 | [0.8240, 0.8251] | 0.7400 | 0.0015 | [0.7371, 0.7426] | 0.7525 | 0.0033 | [0.7472, 0.7586] | 0.7462 | 0.0009 | [0.7448, 0.7480] |
| GCN | 1e-2 | 65.51 | 0.0312 | [65.4415, 65.5589] | 0.7247 | 0.0003 | [0.7241, 0.7251] | 0.6568 | 0.0020 | [0.6536, 0.6625] | 0.6498 | 0.0063 | [0.6329, 0.6591] | 0.6532 | 0.0022 | [0.6470, 0.6566] |
| GTN | 1e-5 | 67.63 | 0.0347 | [67.5567, 67.6878] | 0.7422 | 0.0003 | [0.7415, 0.7428] | 0.6596 | 0.0016 | [0.6562, 0.6618] | 0.7288 | 0.0055 | [0.7213, 0.7396] | 0.6924 | 0.0016 | [0.6901, 0.6957] |
| GIN | 1e-4 | 63.96 | 0.0266 | [63.9039, 64.0054] | 0.7052 | 0.0003 | [0.7046, 0.7058] | 0.6345 | 0.0020 | [0.6322, 0.6402] | 0.6586 | 0.0075 | [0.6365, 0.6668] | 0.6463 | 0.0026 | [0.6383, 0.6490] |

Altogether, the bootstrap-based robustness analysis confirms that AlphaFold2 embeddings are highly stable and reliable input features for protein-protein interaction prediction. These findings support their inclusion as a core component in the DeepDrug Protein Embedding Database (DPEB) and justify their use in downstream tasks requiring consistency and generalizability. Overall, the use of bootstrapped test sets enhances the rigor of our evaluation, ensuring that the observed model differences are not artifacts

© The Author 2025. Published by Oxford University Press. All rights reserved. For permissions, please e-mail: journals.permissions@oup.com

**Supplementary Table 2.** Performance of GNN Models with **BioEmbedding** Embeddings.

| Model | LR | Acc | Acc Std | Acc CI | AUC | AUC Std | AUC CI | Prec | Prec Std | Prec CI | Recall | Recall Std | Recall CI | F1 | F1 Std | F1 CI |
|---|---|---|---|---|---|---|---|---|---|---|---|---|---|---|---|---|
| SAGE | 1e-3 | **79.10** | 0.0267 | [79.0590, 79.1489] | **0.8735** | 0.0002 | [0.8731, 0.8740] | **0.7956** | 0.0015 | [0.7938, 0.7996] | **0.7832** | 0.0025 | [0.7763, 0.7863] | **0.7894** | 0.0006 | [0.7877, 0.7902] |
| GAT | 1e-4 | 75.58 | 0.0253 | [75.5274, 75.6287] | 0.8387 | 0.0002 | [0.8382, 0.8391] | 0.7578 | 0.0018 | [0.7541, 0.7617] | 0.7520 | 0.0034 | [0.7445, 0.7592] | 0.7549 | 0.0009 | [0.7530, 0.7567] |
| GCN | 1e-3 | 66.47 | 0.0302 | [66.4209, 66.5337] | 0.7312 | 0.0003 | [0.7306, 0.7319] | 0.6487 | 0.0010 | [0.6468, 0.6511] | 0.7186 | 0.0037 | [0.7103, 0.7251] | 0.6819 | 0.0011 | [0.6795, 0.6842] |
| GTN | 1e-5 | 71.58 | 0.0311 | [71.5205, 71.6417] | 0.7935 | 0.0003 | [0.7930, 0.7941] | 0.7192 | 0.0016 | [0.7161, 0.7215] | 0.7081 | 0.0036 | [0.7034, 0.7150] | 0.7136 | 0.0011 | [0.7119, 0.7157] |
| GIN | 1e-4 | 63.92 | 0.0285 | [63.8621, 63.9719] | 0.7031 | 0.0003 | [0.7025, 0.7036] | 0.6323 | 0.0017 | [0.6292, 0.6355] | 0.6654 | 0.0070 | [0.6530, 0.6774] | 0.6484 | 0.0024 | [0.6441, 0.6527] |

**Supplementary Table 3.** Performance of GNN Models with **ESM-2** Embeddings.

| Model | LR | Acc | Acc Std | Acc CI | AUC | AUC Std | AUC CI | Prec | Prec Std | Prec CI | Recall | Recall Std | Recall CI | F1 | F1 Std | F1 CI |
|---|---|---|---|---|---|---|---|---|---|---|---|---|---|---|---|---|
| SAGE | 1e-3 | **76.05** | 0.0258 | [75.9973, 76.0986] | **0.8444** | 0.0002 | [0.8439, 0.8448] | **0.7596** | 0.0024 | [0.7555, 0.7653] | **0.7622** | 0.0046 | [0.7520, 0.7701] | **0.7609** | 0.0011 | [0.7584, 0.7630] |
| GAT | 1e-4 | 73.01 | 0.0283 | [72.9503, 73.0529] | 0.8075 | 0.0003 | [0.8069, 0.8080] | 0.7279 | 0.0010 | [0.7256, 0.7293] | 0.7348 | 0.0019 | [0.7321, 0.7395] | 0.7313 | 0.0005 | [0.7305, 0.7326] |
| GCN | 1e-4 | 64.23 | 0.0252 | [64.1839, 64.2779] | 0.7070 | 0.0003 | [0.7065, 0.7075] | 0.6287 | 0.0021 | [0.6260, 0.6340] | 0.6955 | 0.0090 | [0.6735, 0.7082] | 0.6604 | 0.0029 | [0.6531, 0.6646] |
| GTN | 1e-3 | 66.09 | 0.0266 | [66.0397, 66.1419] | 0.7287 | 0.0003 | [0.7283, 0.7292] | 0.6379 | 0.0023 | [0.6341, 0.6411] | 0.7440 | 0.0100 | [0.7318, 0.7596] | 0.6869 | 0.0029 | [0.6833, 0.6915] |
| GIN | 1e-4 | 64.94 | 0.0252 | [64.8862, 64.9877] | 0.7175 | 0.0002 | [0.7170, 0.7180] | 0.6440 | 0.0016 | [0.6421, 0.6481] | 0.6681 | 0.0054 | [0.6549, 0.6739] | 0.6558 | 0.0018 | [0.6513, 0.6578] |

**Supplementary Table 4.** Performance of GNN Models with **ProtVec** Embeddings.

| Model | LR | Acc | Acc Std | Acc CI | AUC | AUC Std | AUC CI | Prec | Prec Std | Prec CI | Recall | Recall Std | Recall CI | F1 | F1 Std | F1 CI |
|---|---|---|---|---|---|---|---|---|---|---|---|---|---|---|---|---|
| SAGE | 1e-3 | **76.53** | 0.0245 | [76.4770, 76.5790] | **0.8494** | 0.0002 | [0.8490, 0.8498] | **0.7636** | 0.0031 | [0.7604, 0.7717] | **0.7686** | 0.0059 | [0.7535, 0.7750] | **0.7661** | 0.0014 | [0.7624, 0.7677] |
| GAT | 1e-4 | 73.51 | 0.0271 | [73.4498, 73.5538] | 0.8149 | 0.0003 | [0.8144, 0.8153] | 0.7311 | 0.0016 | [0.7257, 0.7328] | 0.7437 | 0.0034 | [0.7398, 0.7554] | 0.7373 | 0.0009 | [0.7362, 0.7402] |
| GCN | 1e-4 | 63.14 | 0.0281 | [63.0892, 63.1922] | 0.6964 | 0.0003 | [0.6960, 0.6971] | 0.6240 | 0.0012 | [0.6231, 0.6262] | 0.6612 | 0.0049 | [0.6532, 0.6648] | 0.6420 | 0.0018 | [0.6392, 0.6434] |
| GTN | 1e-5 | 67.22 | 0.0306 | [67.1591, 67.2929] | 0.7408 | 0.0003 | [0.7403, 0.7415] | 0.6562 | 0.0018 | [0.6517, 0.6580] | 0.7237 | 0.0061 | [0.7176, 0.7397] | 0.6883 | 0.0018 | [0.6863, 0.6930] |
| GIN | 1e-4 | 61.00 | 0.0268 | [60.9438, 61.0562] | 0.5742 | 0.0004 | [0.5735, 0.5749] | 0.6820 | 0.0023 | [0.6783, 0.6848] | 0.4123 | 0.0036 | [0.4078, 0.4183] | 0.5139 | 0.0021 | [0.5110, 0.5176] |

of a particular data split. These findings guide more informed decisions in selecting embedding sources and GNN architectures for protein-protein interaction prediction within the DPEB framework.

## McNemar Test on Full Test Data

To statistically compare model performance across different embedding types, we conducted a comprehensive McNemar's test analysis using full test set predictions from each embedding–model configuration. Unlike the bootstrap-based performance evaluations used earlier, this test assesses whether two embeddings yield significantly different prediction outcomes when used within the same model architecture on the same test data.

We implemented a custom evaluation pipeline in Python (see supplementary code) using the `statsmodels` library. For each model (e.g. SAGE, GAT, GCN, GTN, GIN), we generated binary predictions over the test set using four embedding types: AlphaFold2, ESM-2, BioEmbedding, and ProtVec. Predictions and ground truth labels were saved in '.npz' format and loaded for pairwise comparisons.

For each unique pair of embeddings, we constructed a $2 \times 2$ contingency table. These values formed the input to McNemar's test, which we ran using the chi-squared approximation (with continuity correction) when $b + c \geq 25$, and the exact binomial test otherwise. Additionally, we computed an effect size defined as $|b - c|/(b + c)$ to quantify the degree of discrepancy in performance between the two embeddings.

To ensure reliability and traceability, each result—including the contingency values, test statistic, $p$-value, and significance interpretation—was printed and stored in dedicated log files.

We adopted a standard significance threshold of $\alpha = 0.05$, corresponding to a chi-squared critical value of $\chi^2 > 3.84$ with one degree of freedom. Supplementary Tables 5 through 9 summarize the McNemar test results across all pairs of embedding types for each model. A "Yes" in the "Significant?" column indicates that the performance difference between the two embeddings is statistically significant for that model. These tests provide an additional layer of rigor to support the observed trends in model performance across embedding types.

**Supplementary Table 5.** Pairwise McNemar Test Comparisons of Embedding Types (**SAGE** Model)

| **Comparison** | **Both Correct** | **Only Model 1 Correct** | **Only Model 2 Correct** | **Both Incorrect** | **$\chi^2$ Statistic** | **Significant?** |
|---|---|---|---|---|---|---|
| AlphaFold2 vs ESM-2 | 1,442,813 | 215,705 | 285,238 | 235,038 | 9651.20 | Yes |
| AlphaFold2 vs BioEmbedding | 1,439,718 | 218,800 | 283,645 | 236,631 | 8368.57 | Yes |
| AlphaFold2 vs ProtVec | 1,419,002 | 239,516 | 248,548 | 271,728 | 167.11 | Yes |
| ESM-2 vs BioEmbedding | 1,508,816 | 219,235 | 214,547 | 236,196 | 50.64 | Yes |
| ESM-2 vs ProtVec | 1,449,794 | 278,257 | 217,756 | 232,987 | 7379.34 | Yes |
| BioEmbedding vs ProtVec | 1,446,707 | 276,656 | 220,843 | 234,588 | 6261.28 | Yes |

Across the five GNN models evaluated, most embedding pairs exhibited statistically significant differences in prediction behavior (Supplementary Tables 5 through 9). Specifically, BioEmbedding consistently differed from ProtVec and ESM-2, suggesting meaningful variation in the way these embeddings encode protein features. Notably, in the GIN model, the comparison between AlphaFold2 and BioEmbedding was not statistically significant ($\chi^2 = 1.60$), implying similar performance in that specific architecture. In all other cases, AlphaFold2 demonstrated statistically distinguishable behavior compared to at least one other embedding type. These findings confirm that model performance variability across embeddings is not merely due to random fluctuations but reflects systematic differences in the learned protein representations.

**Supplementary Table 6.** Pairwise McNemar Test Comparisons of Embedding Types (**GAT** Model)

| Comparison | Both Correct | Only Model 1 Correct | Only Model 2 Correct | Both Incorrect | $\chi^2$ Statistic | Significant? |
|---|---|---|---|---|---|---|
| AlphaFold2 vs ESM-2 | 1,374,300 | 246,743 | 285,708 | 272,043 | 2851.33 | Yes |
| AlphaFold2 vs BioEmbedding | 1,354,872 | 266,171 | 291,864 | 265,887 | 1182.86 | Yes |
| AlphaFold2 vs ProtVec | 1,333,595 | 287,448 | 267,891 | 289,860 | 688.66 | Yes |
| ESM-2 vs BioEmbedding | 1,415,993 | 244,015 | 230,743 | 288,043 | 370.97 | Yes |
| ESM-2 vs ProtVec | 1,365,426 | 294,582 | 236,060 | 282,726 | 6453.89 | Yes |
| BioEmbedding vs ProtVec | 1,359,672 | 287,064 | 241,814 | 290,244 | 3871.35 | Yes |

**Supplementary Table 7.** Pairwise McNemar Test Comparisons of Embedding Types (**GCN** Model)

| Comparison | Both Correct | Only Model 1 Correct | Only Model 2 Correct | Both Incorrect | $\chi^2$ Statistic | Significant? |
|---|---|---|---|---|---|---|
| AlphaFold2 vs ESM-2 | 1,181,391 | 245,753 | 288,951 | 462,699 | 3489.75 | Yes |
| AlphaFold2 vs BioEmbedding | 1,072,193 | 354,951 | 376,031 | 375,619 | 607.85 | Yes |
| AlphaFold2 vs ProtVec | 1,119,551 | 307,593 | 256,083 | 495,567 | 4706.92 | Yes |
| ESM-2 vs BioEmbedding | 1,128,303 | 342,039 | 319,921 | 388,531 | 738.96 | Yes |
| ESM-2 vs ProtVec | 1,153,455 | 316,887 | 222,179 | 486,273 | 16638.81 | Yes |
| BioEmbedding vs ProtVec | 1,052,983 | 395,241 | 322,651 | 407,919 | 7339.77 | Yes |

**Supplementary Table 8.** Pairwise McNemar Test Comparisons of Embedding Types (**GTN** Model)

| Comparison | Both Correct | Only Model 1 Correct | Only Model 2 Correct | Both Incorrect | $\chi^2$ Statistic | Significant? |
|---|---|---|---|---|---|---|
| AlphaFold2 vs ESM-2 | 1,118,732 | 354,635 | 349,094 | 356,333 | 43.61 | Yes |
| AlphaFold2 vs BioEmbedding | 1,183,200 | 290,167 | 376,408 | 329,019 | 11157.54 | Yes |
| AlphaFold2 vs ProtVec | 1,158,220 | 315,147 | 306,406 | 399,021 | 122.90 | Yes |
| ESM-2 vs BioEmbedding | 1,160,167 | 307,659 | 399,441 | 311,527 | 11913.10 | Yes |
| ESM-2 vs ProtVec | 1,108,838 | 358,988 | 355,788 | 355,180 | 14.32 | Yes |
| BioEmbedding vs ProtVec | 1,169,287 | 390,321 | 295,339 | 323,847 | 13157.24 | Yes |

**Supplementary Table 9.** Pairwise McNemar Test Comparisons of Embedding Types (**GIN** Model)

| Comparison | Both Correct | Only Model 1 Correct | Only Model 2 Correct | Both Incorrect | $\chi^2$ Statistic | Significant? |
|---|---|---|---|---|---|---|
| AlphaFold2 vs ESM-2 | 1,143,770 | 249,678 | 340,947 | 444,399 | 14103.45 | Yes |
| AlphaFold2 vs BioEmbedding | 1,163,921 | 229,527 | 228,669 | 556,677 | 1.60 | No |
| AlphaFold2 vs ProtVec | 919,068 | 474,380 | 409,981 | 375,365 | 4689.38 | Yes |
| ESM-2 vs BioEmbedding | 1,152,453 | 332,264 | 240,137 | 453,940 | 14827.37 | Yes |
| ESM-2 vs ProtVec | 1,021,214 | 463,503 | 307,835 | 386,242 | 31415.82 | Yes |
| BioEmbedding vs ProtVec | 928,896 | 463,694 | 400,153 | 386,051 | 4673.67 | Yes |

## Detailed Results of Use Case 3: Protein Family Analysis Through Clustering and Classification

In the main paper (Section "Use Case 3: Protein Family Analysis Through Clustering and Classification"), we introduced a two-phase workflow for evaluating the biological relevance of AlphaFold2-derived structural embeddings through both unsupervised clustering and supervised classification of 2,399 human proteins across 17 protein families. Here, we extend those results with comparative evaluations across ProtVec, ESM-2, and BioEmbedding embeddings in addition to AlphaFold2. The methodology for Transformer-based refinement of AlphaFold2 embeddings is described in detail in the main manuscript.

We applied the same Transformer encoder to create refined versions of BioEmbedding, ESM-2, and ProtVec embeddings using identical preprocessing and evaluation procedures. This includes L2-normalization, PCA dimensionality reduction (to retain 95% variance), K-means clustering, and evaluation via standard classification metrics as downstream task. To assess how well each embedding space aligns with known protein family structure, we performed K-means clustering on both raw and Transformer-refined embeddings. For refined embeddings, labels are used only during Transformer training; the clustering itself is fully unsupervised and serves as exploratory analysis and visualization of how supervision shapes the latent space structure. Cluster–label agreement was evaluated using permutation-invariant metrics, including Adjusted Rand Index (ARI), Normalized Mutual Information (NMI), and homogeneity–completeness–V-measure scores. Importantly, clustering is performed without access to labels, and these metrics are permutation-invariant to cluster labels. This ensures that results reflect the geometric structure of the embedding space rather than label memorization.

Supplementary Table 10 summarizes these results across AlphaFold2, BioEmbedding, ESM-2, and ProtVec representations. Clustering and evaluation are performed in an unsupervised manner: K-means does not use labels, and clustering metrics (ARI, NMI, homogeneity, completeness, and V-measure) are permutation-invariant and computed post hoc to assess cluster–label alignment, ensuring that no label information is used during clustering. While raw embeddings show moderate correspondence with protein family labels, Transformer-refined embeddings consistently achieve near-perfect agreement across all metrics. These results indicate that supervised refinement substantially reshapes the embedding geometry, providing highly compact and well-separated family-level clusters.

**Supplementary Table 10.** Clustering quality comparison of raw and refined protein embeddings using K-means. Refined embeddings are obtained via supervised transformer-based representation learning and show substantially improved alignment with protein family labels across all metrics.

| Embedding | Raw Embeddings | | | | | Refined Embeddings | | | | |
|---|---|---|---|---|---|---|---|---|---|---|
| | ARI | NMI | Homogeneity | Completeness | V-measure | ARI | NMI | Homogeneity | Completeness | V-measure |
| AlphaFold2 | 0.2903 | 0.4627 | 0.4971 | 0.4328 | 0.4627 | **0.9754** | **0.9283** | **0.9296** | **0.9270** | **0.9283** |
| Bio | 0.3266 | 0.5333 | 0.5805 | 0.4932 | 0.5333 | **0.9986** | **0.9945** | **0.9944** | **0.9946** | **0.9945** |
| ESM-2 | 0.4027 | 0.5306 | 0.5606 | 0.5037 | 0.5306 | **0.9991** | **0.9962** | **0.9962** | **0.9963** | **0.9962** |
| ProtVec | 0.2652 | 0.4535 | 0.4766 | 0.4324 | 0.4535 | **0.9812** | **0.9320** | **0.9318** | **0.9322** | **0.9320** |

Table 6 from the main paper reports the classification performance of raw versus Transformer-refined AlphaFold2 embeddings across different classifiers including Naive Bayes Decision Tree, KNN, Random Forest, and a FCN-based classifier. As shown, refined embeddings yielded consistent improvements in accuracy, precision, recall, and F1-score across nearly all classifiers, indicating that supervised refinement can significantly enhance the separability of family-level protein features.

Supplementary Figure 1 presents t-SNE visualizations of K-means clusters on both raw and refined embeddings for all four embedding types. Each subplot (a–h) corresponds to a unique combination of embedding type and refinement stage. Consistent with our observations in the main paper (Figure 3), the refined embeddings across all modalities demonstrate enhanced cluster compactness and clearer boundaries between protein families. This visual evidence further supports the classification results, illustrating that deep refinement models can amplify latent biological signals within diverse embedding spaces.

Taken together, the results in Supplementary Tables 11 through 13 demonstrate that the Transformer-based refinement strategy developed for AlphaFold2 embeddings generalizes effectively to other protein representation modalities. This reinforces the potential of the DPEB framework not only for structural interpretation but also for multimodal integration and functional protein analysis across diverse bioinformatics tasks.

**Supplementary Table 11.** Classification performance comparison of raw vs. refined BioEmbeddings across multiple models. Refined embeddings show consistently higher performance across all metrics.

| Model | Raw Embeddings | | | | Refined Embeddings | | | |
|---|---|---|---|---|---|---|---|---|
| | Accuracy | Precision | Recall | F1-Score | Accuracy | Precision | Recall | F1-Score |
| Naive Bayes | 0.8243 | 0.8523 | 0.8243 | 0.8320 | 0.9833 | 0.9841 | 0.9833 | 0.9831 |
| Decision Tree | 0.7636 | 0.8330 | 0.7636 | 0.7680 | 0.8661 | 0.9161 | 0.8661 | 0.8753 |
| FCN | **0.9226** | **0.9443** | **0.9226** | **0.9213** | 0.9895 | 0.9901 | 0.9895 | 0.9894 |
| KNN | 0.9100 | 0.9276 | 0.9100 | 0.9111 | 0.9854 | 0.9857 | 0.9854 | 0.9851 |
| Random Forest | 0.9644 | 0.9664 | 0.9644 | 0.9635 | 0.9874 | 0.9880 | 0.9874 | 0.9872 |

**Supplementary Table 12.** Performance comparison of raw vs. refined ESM-2 embeddings across different models. Refined ESM-2 embeddings achieve consistently higher accuracy, precision, recall, and F1-score.

| Model | Raw Embeddings | | | | Refined Embeddings | | | |
|---|---|---|---|---|---|---|---|---|
| | Accuracy | Precision | Recall | F1-Score | Accuracy | Precision | Recall | F1-Score |
| Random Forest | **0.9732** | **0.9737** | **0.9732** | **0.9729** | 0.9918 | 0.9924 | 0.9918 | 0.9917 |
| KNN | 0.9320 | 0.9460 | 0.9320 | 0.9327 | 0.9918 | 0.9924 | 0.9918 | 0.9917 |
| Naive Bayes | 0.8722 | 0.9167 | 0.8722 | 0.8867 | 0.9897 | 0.9902 | 0.9897 | 0.9898 |
| Decision Tree | 0.7134 | 0.7803 | 0.7134 | 0.7236 | 0.8701 | 0.9107 | 0.8701 | 0.8700 |
| FCN | 0.9691 | 0.9718 | 0.9691 | 0.9674 | 0.9918 | 0.9927 | 0.9918 | 0.991 |

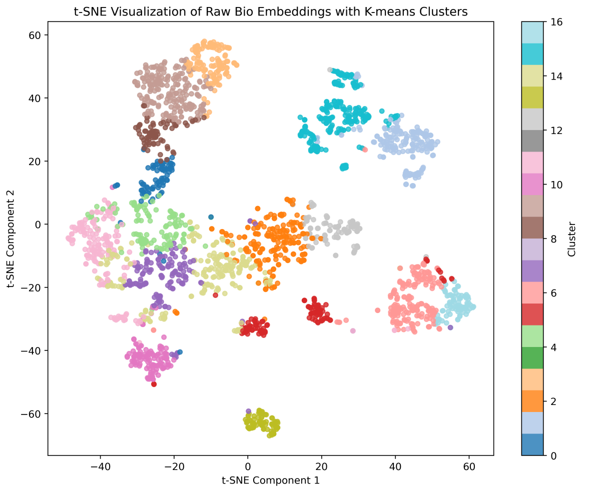


(a) Raw BioEmbeddings

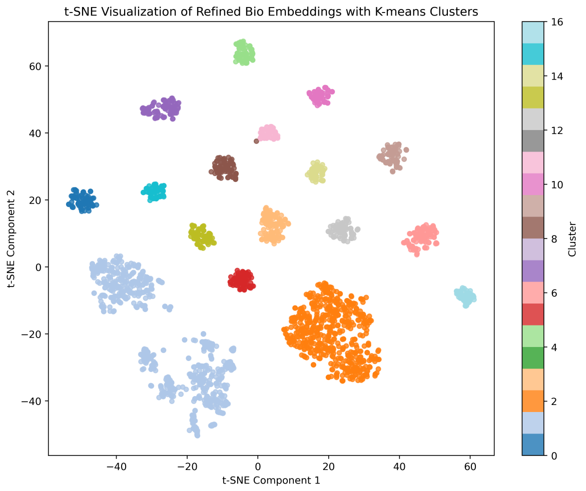


(b) Refined BioEmbeddings

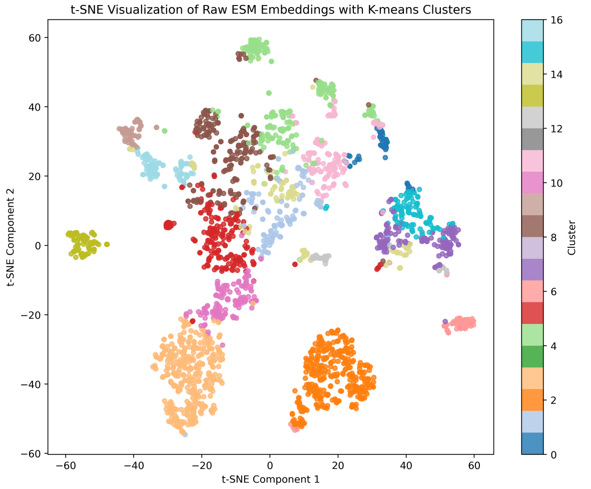


(c) Raw ESM-2 embeddings

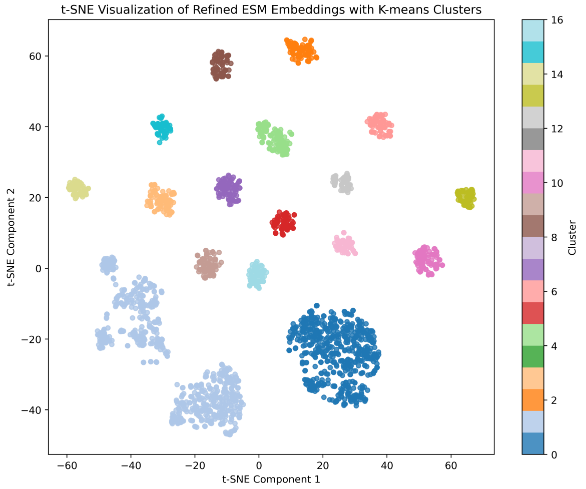


(d) Refined ESM-2 embeddings

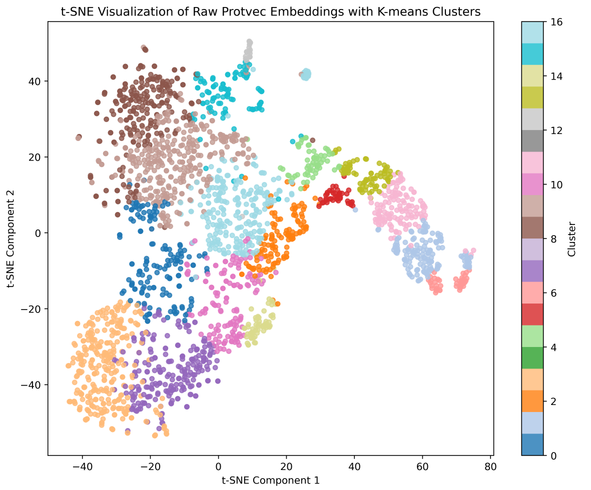


(e) Raw ProtVec embeddings

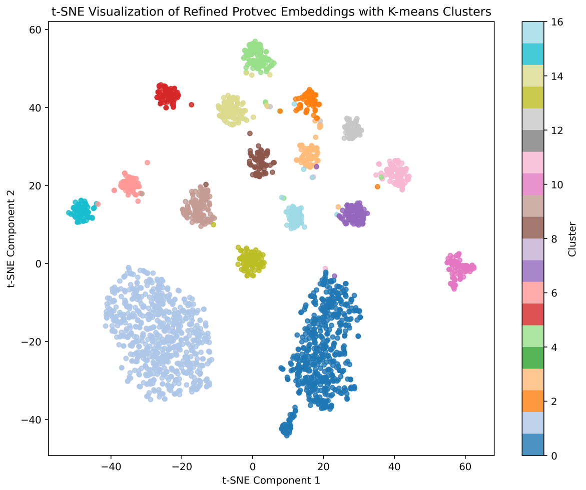


(f) Refined ProtVec embeddings

**Supplementary Fig. 1.** t-SNE visualizations comparing raw and refined protein embeddings using K-means clustering across three embedding types: BioEmbedding, ESM-2, and ProtVec. Each protein is colored by its assigned cluster. Refinement improves cluster separation and compactness. AlphaFold2 comparisons are presented in the main paper.

**Supplementary Table 13.** Performance comparison of raw vs. refined ProtVec embeddings across different models. Refined embeddings consistently show improved performance in most classifiers.

| Model | Raw Embeddings | | | | Refined Embeddings | | | |
|---|---|---|---|---|---|---|---|---|
| | Accuracy | Precision | Recall | F1-Score | Accuracy | Precision | Recall | F1-Score |
| Naive Bayes | 0.6660 | 0.7172 | 0.6660 | 0.6760 | 0.8103 | 0.8368 | 0.8103 | 0.8190 |
| Decision Tree | 0.5979 | 0.6297 | 0.5979 | 0.6052 | 0.7072 | 0.7153 | 0.7072 | 0.7027 |
| FCN | 0.6990 | 0.7045 | 0.6990 | 0.6610 | 0.8227 | 0.8221 | 0.8227 | 0.8189 |
| KNN | 0.6866 | 0.7744 | 0.6866 | 0.6808 | 0.8289 | 0.8267 | 0.8289 | 0.8249 |
| Random Forest | **0.7835** | **0.7835** | **0.7835** | **0.7745** | 0.8289 | 0.8293 | 0.8289 | 0.8262 |

## Dataset Accessibility

DPEB provides protein embeddings in four types: AlphaFold2, ProtVec, ESM-2, and BioEmbedding. The database includes raw residue-level embeddings as well as aggregated protein-level embeddings, allowing users to choose between detailed sequence features or summarized representations depending on the application.

We are hosting the DeepDrug Protein Embeddings Bank (DPEB) on the Amazon Web Services (AWS) Open Data Program.By making DPEB openly accessible via AWS, we aim to facilitate reproducibility, accelerate downstream applications in drug development, and promote systems biology research. The dataset can be accessed from the DPEB AWS S3 bucket: https://registry.opendata.aws/deepdrug-dpeb/. To simplify data exploration and download, we provide a Google Colab notebook with ready-to-use scripts for interacting with the AWS S3 bucket (deepdrug-dpeb). The Colab notebook and step-by-step download instructions are available here: DPEB Access and Download Link.

### Dataset Repository Structure

The DeepDrug Protein Embeddings Bank (DPEB) repository is organized as follows:

```
deepdrug-dpeb/
|
|-- dpeb_aggreagated_embeddings_all_in_one.csv
|
|-- AlphaFold-2/
|   |-- All_ePPI_AlphaFold2_Embeddings_np_v1.3.rar
|   `-- eppi_alphafold_aggregated_embeddings.csv
|
|-- ESM-2/
|   |-- esm2_dict_embeddings.rar
|   `-- ProteinID_proteinSEQ_ESM_emb.csv
|
|-- ProtVec/
|   |-- protvec_dict_embeddings.rar
|   `-- protvec_aggregated_embeddings.csv
|
`-- BioEmbedding/
    |-- All_ePPI_Bio_Embeddings_np.rar
    `-- bio_embeddings_ePPI.csv
```

### File Descriptions

The DeepDrug Protein Embeddings Bank (DPEB) contains both raw per-protein embeddings and pre-aggregated metadata to accommodate different levels of analysis. To help users select the most appropriate files for their workflows, we provide the following descriptions and recommendations.

- **.rar files:** Archives containing individual .npy embedding files for each protein.
- **.csv files:** Metadata files containing UniProt IDs, amino acid sequences, and aggregated embeddings.
- **dpeb_aggreagated_embeddings_all_in_one.csv:** Combined metadata and aggregated embeddings for all proteins across all embedding types.

All raw embeddings are structured as numpy (.npy) files containing protein information. The data is organized consistently with three key features: *protein* (string identifier), *fasta* (raw protein sequence) and *embeddings* (encoded vector representations stored as strings). The detailed dataset directory structure, data loading and usage instructions are provided with code in the Github repository available at: https://github.com/deepdrugai/DPEB.

## User Suggestions – When to Use Which File

- **Use the .rar archives** if you need individual per-protein embeddings and wish to analyze or model proteins separately (e.g., for custom downstream tasks or when working with raw embedding matrices).
- **Use the .csv metadata files in each directory** if you want aggregated embeddings (e.g., averaged across residues) with quick access to UniProt IDs and sequences. These are well-suited for prototyping, graph construction, or machine learning tasks that do not require residue-level resolution.
- **Use dpeb_aggreagated_embeddings_all_in_one.csv** if you need a single file containing aggregated embeddings of all modalities for all proteins. This file is recommended for benchmarking, tabular machine learning, or analyses requiring a unified multimodal representation. It provides separate columns for AlphaFold2, BioEmbedding, ESM-2, and ProtVec embeddings, enabling flexible selection, combination, or comparison across embedding types.

Taken together, these complementary file formats allow users to select between fine-grained embeddings for customized analysis and compact metadata tables for efficient large-scale workflows.

## Programmatic Access via Python (Colab Notebook)

The **DeepDrug Protein Embeddings Bank (DPEB)** is hosted via the AWS Open Data Sponsorship Program and is accessible without authentication through Amazon S3 under the bucket name `deepdrug-dpeb`. Programmatic access is available using the AWS Python SDK (`boto3`) in anonymous mode. The following example illustrates how to list top-level directories and download specific files:

```
import boto3
from botocore.config import Config
from botocore import UNSIGNED

s3 = boto3.client('s3', config=Config(signature_version=UNSIGNED))
bucket_name = 'deepdrug-dpeb'

# List top-level folders
response = s3.list_objects_v2(Bucket=bucket_name, Delimiter='/')
for prefix in response.get('CommonPrefixes', []):
    print(prefix['Prefix'])

# Download a specific file
file_key = 'AlphaFold-2/eppi_alphafold_aggregated_embeddings.csv'
s3.download_file(bucket_name, file_key, 'eppi_alphafold_aggregated_embeddings.csv',
                 ExtraArgs={'RequestPayer': 'requester'})
```

Note: AWS requires that the `RequestPayer='requester'` argument be explicitly included for downloads from public buckets in the Open Data program.

## Programmatic Access via AWS CLI

In addition to Python `boto3` examples, users can also directly download files from the DeepDrug Protein Embeddings Bank (DPEB) using the AWS Command Line Interface (CLI). This method is recommended for large-scale transfers or automated workflows. No AWS account or credentials are required, since the dataset is hosted under the AWS Open Data Program.

Install (Linux example):

```
curl "https://awscli.amazonaws.com/awscli-exe-linux-x86_64.zip" -o "awscliv2.zip"
unzip awscliv2.zip
sudo ./aws/install
```

Download example:

```
aws s3 cp s3://deepdrug-dpeb/ProtVec/protvec_aggregated_embeddings.csv . --no-sign-request
```

This fetches the aggregated ProtVec embeddings into the current directory. The same command can be adapted for any path under `s3://deepdrug-dpeb/`.

## Ablation Study

This ablation study evaluates the contribution of different protein embedding combinations in the DPEB framework. We systematically analyze pairwise, triple, and full embedding combinations constructed from AlphaFold2, BioEmbedding (Bio), ESM-2, and ProtVec representations.

For each embedding combination, the learning rate was selected from {1e-3, 1e-4, 1e-5} based on validation performance. All results reported below (Supplementary Table 14 through Supplementary Table 18) correspond to the best-performing learning rate for each configuration. Evaluation is conducted using the DeepDrug protocol and reported on a fixed test set.

**Supplementary Table 14.** Robustness performance of the **SAGE** model with pairwise, triple, and full embedding combinations using bootstrapping (100 iterations). For each configuration, results correspond to the best-tuned learning rate selected via the same testset.

| Embedding | LR | Acc | Acc Std | Acc CI | AUC | AUC Std | AUC CI | Prec | Prec Std | Prec CI | Recall | Recall Std | Recall CI | F1 | F1 Std | F1 CI |
|---|---|---|---|---|---|---|---|---|---|---|---|---|---|---|---|---|
| AlphaFold2 + Bio | 1e-3 | 78.84 | 0.0304 | [78.77, 78.90] | 0.8714 | 0.0003 | [0.8708, 0.8720] | 0.7934 | 0.0009 | [0.7921, 0.7956] | 0.7799 | 0.0015 | [0.7765, 0.7821] | 0.7866 | 0.0004 | [0.7857, 0.7874] |
| AlphaFold2 + ESM-2 | 1e-3 | 78.85 | 0.0307 | [78.79, 78.92] | 0.8716 | 0.0003 | [0.8710, 0.8721] | 0.7956 | 0.0016 | [0.7938, 0.7996] | 0.7767 | 0.0026 | [0.7695, 0.7798] | 0.7860 | 0.0006 | [0.7844, 0.7868] |
| AlphaFold2 + ProtVec | 1e-3 | 76.61 | 0.0274 | [76.56, 76.66] | 0.8495 | 0.0002 | [0.8490, 0.8499] | 0.7700 | 0.0013 | [0.7675, 0.7735] | 0.7588 | 0.0024 | [0.7523, 0.7627] | 0.7643 | 0.0007 | [0.7627, 0.7655] |
| Bio + ESM-2 | 1e-3 | **79.29** | 0.0272 | [79.23, 79.34] | **0.8751** | 0.0002 | [0.8745, 0.8756] | **0.8034** | 0.0019 | [0.8003, 0.8061] | 0.7756 | 0.0029 | [0.7717, 0.7796] | **0.7893** | 0.0006 | [0.7883, 0.7902] |
| Bio + ProtVec | 1e-3 | 78.77 | 0.0289 | [78.71, 78.82] | 0.8707 | 0.0003 | [0.8702, 0.8712] | 0.7955 | 0.0022 | [0.7902, 0.7985] | 0.7744 | 0.0035 | [0.7700, 0.7835] | 0.7848 | 0.0008 | [0.7837, 0.7865] |
| ESM-2 + ProtVec | 1e-3 | 78.78 | 0.0286 | [78.72, 78.84] | 0.8707 | 0.0002 | [0.8702, 0.8711] | 0.7973 | 0.0011 | [0.7948, 0.7995] | 0.7719 | 0.0018 | [0.7687, 0.7763] | 0.7844 | 0.0005 | [0.7835, 0.7854] |
| AlphaFold2 + Bio + ESM-2 | 1e-3 | 79.24 | 0.0289 | [79.18, 79.28] | 0.8748 | 0.0003 | [0.8742, 0.8753] | 0.8013 | 0.0029 | [0.7977, 0.8065] | 0.7777 | 0.0046 | [0.7690, 0.7832] | 0.7893 | 0.0010 | [0.7874, 0.7906] |
| AlphaFold2 + Bio + ProtVec | 1e-3 | 78.68 | 0.0300 | [78.62, 78.74] | 0.8701 | 0.0003 | [0.8695, 0.8706] | 0.7923 | 0.0019 | [0.7889, 0.7962] | 0.7775 | 0.0031 | [0.7716, 0.7828] | 0.7848 | 0.0007 | [0.7835, 0.7860] |
| AlphaFold2 + ESM-2 + ProtVec | 1e-3 | 78.68 | 0.0275 | [78.63, 78.73] | 0.8698 | 0.0003 | [0.8692, 0.8704] | 0.7917 | 0.0023 | [0.7879, 0.7969] | 0.7785 | 0.0040 | [0.7695, 0.7851] | 0.7850 | 0.0009 | [0.7829, 0.7867] |
| Bio + ESM-2 + ProtVec | 1e-3 | 79.10 | 0.0259 | [79.05, 79.15] | 0.8736 | 0.0002 | [0.8730, 0.8741] | 0.7999 | 0.0019 | [0.7969, 0.8038] | 0.7761 | 0.0031 | [0.7701, 0.7811] | 0.7878 | 0.0007 | [0.7864, 0.7890] |
| AlphaFold2 + ProtVec + Bio + ESM-2 | 1e-3 | 79.09 | 0.0281 | [79.03, 79.14] | 0.8739 | 0.0002 | [0.8733, 0.8744] | 0.7979 | 0.0036 | [0.7918, 0.8063] | 0.7792 | 0.0058 | [0.7661, 0.7893] | 0.7884 | 0.0013 | [0.7854, 0.7906] |

**Supplementary Table 15.** Robustness performance of the **GAT** model with pairwise, triple, and full embedding combinations using bootstrapping (100 iterations). For each configuration, results correspond to the best-tuned learning rate selected via the same testset.

| Embedding | LR | Acc | Acc Std | Acc CI | AUC | AUC Std | AUC CI | Prec | Prec Std | Prec CI | Recall | Recall Std | Recall CI | F1 | F1 Std | F1 CI |
|---|---|---|---|---|---|---|---|---|---|---|---|---|---|---|---|---|
| AlphaFold2 + Bio | 1e-4 | 75.39 | 0.0300 | [75.32, 75.44] | 0.8375 | 0.0003 | [0.8370, 0.8380] | 0.7576 | 0.0019 | [0.7532, 0.7604] | 0.7467 | 0.0036 | [0.7419, 0.7552] | 0.7521 | 0.0009 | [0.7506, 0.7542] |
| AlphaFold2 + ESM-2 | 1e-4 | 75.27 | 0.0277 | [75.22, 75.32] | 0.8349 | 0.0003 | [0.8344, 0.8354] | 0.7518 | 0.0025 | [0.7483, 0.7557] | 0.7546 | 0.0050 | [0.7466, 0.7607] | 0.7532 | 0.0013 | [0.7511, 0.7548] |
| AlphaFold2 + ProtVec | 1e-4 | 73.10 | 0.0301 | [73.04, 73.16] | 0.8098 | 0.0003 | [0.8092, 0.8104] | 0.7199 | 0.0011 | [0.7187, 0.7225] | 0.7562 | 0.0024 | [0.7508, 0.7586] | 0.7376 | 0.0007 | [0.7360, 0.7385] |
| Bio + ESM-2 | 1e-4 | **75.55** | 0.0263 | [75.49, 75.59] | **0.8385** | 0.0003 | [0.8379, 0.8389] | 0.7566 | 0.0012 | [0.7539, 0.7582] | 0.7533 | 0.0023 | [0.7503, 0.7600] | 0.7549 | 0.0006 | [0.7539, 0.7567] |
| Bio + ProtVec | 1e-4 | 75.31 | 0.0283 | [75.25, 75.37] | 0.8363 | 0.0003 | [0.8357, 0.8367] | 0.7535 | 0.0016 | [0.7498, 0.7560] | 0.7523 | 0.0030 | [0.7476, 0.7596] | 0.7529 | 0.0008 | [0.7516, 0.7547] |
| ESM-2 + ProtVec | 1e-4 | 75.09 | 0.0274 | [75.04, 75.14] | 0.8336 | 0.0003 | [0.8331, 0.8341] | 0.7523 | 0.0024 | [0.7487, 0.7554] | 0.7481 | 0.0045 | [0.7423, 0.7554] | 0.7502 | 0.0011 | [0.7487, 0.7522] |
| AlphaFold2 + Bio + ESM-2 | 1e-4 | 75.56 | 0.0268 | [75.50, 75.61] | **0.8388** | 0.0003 | [0.8383, 0.8394] | 0.7539 | 0.0009 | [0.7527, 0.7564] | **0.7590** | 0.0016 | [0.7539, 0.7609] | **0.7564** | 0.0005 | [0.7552, 0.7572] |
| AlphaFold2 + Bio + ProtVec | 1e-4 | 75.21 | 0.0295 | [75.16, 75.28] | 0.8350 | 0.0003 | [0.8345, 0.8355] | 0.7517 | 0.0023 | [0.7465, 0.7538] | 0.7531 | 0.0045 | [0.7495, 0.7636] | 0.7524 | 0.0012 | [0.7511, 0.7550] |
| AlphaFold2 + ESM-2 + ProtVec | 1e-4 | 75.13 | 0.0276 | [75.07, 75.18] | 0.8341 | 0.0003 | [0.8335, 0.8346] | 0.7498 | 0.0016 | [0.7476, 0.7524] | 0.7542 | 0.0032 | [0.7494, 0.7587] | 0.7520 | 0.0008 | [0.7506, 0.7533] |
| Bio + ESM-2 + ProtVec | 1e-4 | 75.47 | 0.0270 | [75.41, 75.53] | 0.8373 | 0.0003 | [0.8368, 0.8379] | 0.7551 | 0.0029 | [0.7511, 0.7588] | 0.7540 | 0.0058 | [0.7471, 0.7615] | 0.7545 | 0.0015 | [0.7525, 0.7567] |
| AlphaFold2 + ProtVec + Bio + ESM-2 | 1e-4 | 75.52 | 0.0288 | [75.48, 75.57] | 0.8384 | 0.0003 | [0.8379, 0.8389] | **0.7560** | 0.0014 | [0.7535, 0.7601] | 0.7537 | 0.0027 | [0.7459, 0.7578] | 0.7549 | 0.0007 | [0.7529, 0.7557] |

**Supplementary Table 16.** Robustness performance of the **GCN** model with pairwise, triple, and full embedding combinations using bootstrapping (100 iterations). For each configuration, results correspond to the best-tuned learning rate selected via the same testset.

| Embedding | LR | Acc | Acc Std | Acc CI | AUC | AUC Std | AUC CI | Prec | Prec Std | Prec CI | Recall | Recall Std | Recall CI | F1 | F1 Std | F1 CI |
|---|---|---|---|---|---|---|---|---|---|---|---|---|---|---|---|---|
| AlphaFold2 + Bio | 1e-5 | 62.80 | 0.0273 | [62.74, 62.85] | 0.6899 | 0.0002 | [0.6894, 0.6903] | 0.6179 | 0.0019 | [0.6155, 0.6212] | 0.6707 | 0.0085 | [0.6562, 0.6823] | 0.6432 | 0.0029 | [0.6383, 0.6473] |
| AlphaFold2 + ESM-2 | 1e-5 | 67.05 | 0.0274 | [67.00, 67.09] | 0.7422 | 0.0003 | [0.7417, 0.7427] | 0.6593 | 0.0019 | [0.6551, 0.6620] | 0.7057 | 0.0064 | [0.6970, 0.7193] | 0.6817 | 0.0020 | [0.6790, 0.6859] |
| AlphaFold2 + ProtVec | 1e-3 | 63.50 | 0.0288 | [63.44, 63.55] | 0.6966 | 0.0002 | [0.6961, 0.6970] | 0.6275 | 0.0016 | [0.6244, 0.6303] | 0.6642 | 0.0065 | [0.6539, 0.6750] | 0.6453 | 0.0022 | [0.6417, 0.6490] |
| Bio + ESM-2 | 1e-5 | **67.74** | 0.0277 | [67.69, 67.79] | **0.7468** | 0.0003 | [0.7462, 0.7474] | **0.6646** | 0.0015 | [0.6615, 0.6670] | **0.7162** | 0.0047 | [0.7082, 0.7271] | **0.6895** | 0.0014 | [0.6870, 0.6925] |
| Bio + ProtVec | 1e-5 | 64.19 | 0.0290 | [64.13, 64.24] | 0.7089 | 0.0003 | [0.7084, 0.7093] | 0.6405 | 0.0023 | [0.6364, 0.6444] | 0.6469 | 0.0084 | [0.6346, 0.6593] | 0.6436 | 0.0030 | [0.6391, 0.6480] |
| ESM-2 + ProtVec | 1e-5 | 62.63 | 0.0276 | [62.57, 62.68] | 0.6885 | 0.0002 | [0.6880, 0.6890] | 0.6158 | 0.0033 | [0.6119, 0.6228] | 0.6718 | 0.0151 | [0.6420, 0.6894] | 0.6425 | 0.0052 | [0.6319, 0.6485] |
| AlphaFold2 + Bio + ESM-2 | 1e-4 | 66.29 | 0.0269 | [66.24, 66.34] | 0.7350 | 0.0003 | [0.7345, 0.7355] | 0.6634 | 0.0021 | [0.6610, 0.6681] | 0.6613 | 0.0066 | [0.6464, 0.6681] | 0.6623 | 0.0023 | [0.6571, 0.6647] |
| AlphaFold2 + Bio + ProtVec | 1e-3 | 65.56 | 0.0274 | [65.50, 65.61] | 0.7199 | 0.0003 | [0.7195, 0.7205] | 0.6394 | 0.0031 | [0.6336, 0.6440] | 0.7138 | 0.0126 | [0.6963, 0.7370] | 0.6745 | 0.0039 | [0.6689, 0.6818] |
| AlphaFold2 + ESM-2 + ProtVec | 1e-5 | 63.15 | 0.0299 | [63.09, 63.20] | 0.6952 | 0.0003 | [0.6946, 0.6957] | 0.6254 | 0.0020 | [0.6208, 0.6283] | 0.6560 | 0.0079 | [0.6452, 0.6741] | 0.6403 | 0.0027 | [0.6364, 0.6463] |
| Bio + ESM-2 + ProtVec | 1e-5 | 66.41 | 0.0264 | [66.36, 66.45] | 0.7341 | 0.0003 | [0.7336, 0.7346] | 0.6534 | 0.0026 | [0.6499, 0.6585] | 0.6993 | 0.0090 | [0.6816, 0.7103] | 0.6755 | 0.0028 | [0.6697, 0.6790] |
| AlphaFold2 + ProtVec + Bio + ESM-2 | 1e-5 | 64.91 | 0.0281 | [64.86, 64.96] | 0.7173 | 0.0003 | [0.7168, 0.7178] | 0.6457 | 0.0021 | [0.6425, 0.6493] | 0.6610 | 0.0074 | [0.6492, 0.6714] | 0.6532 | 0.0025 | [0.6491, 0.6568] |

**Supplementary Table 17.** Robustness performance of the **GTN** model with pairwise, triple, and full embedding combinations using bootstrapping (100 iterations). For each configuration, results correspond to the best-tuned learning rate selected via the same testset.

| **Embedding** | **LR** | **Acc** | **Acc Std** | **Acc CI** | **AUC** | **AUC Std** | **AUC CI** | **Prec** | **Prec Std** | **Prec CI** | **Recall** | **Recall Std** | **Recall CI** | **F1** | **F1 Std** | **F1 CI** |
|---|---|---|---|---|---|---|---|---|---|---|---|---|---|---|---|---|
| AlphaFold2 + Bio | 1e-4 | 72.58 | 0.0277 | [72.52, 72.63] | 0.8047 | 0.0003 | [0.8042, 0.8052] | 0.7341 | 0.0029 | [0.7300, 0.7386] | 0.7079 | 0.0059 | [0.6981, 0.7155] | 0.7208 | 0.0017 | [0.7178, 0.7229] |
| AlphaFold2 + ESM-2 | 1e-4 | 73.37 | 0.0325 | [73.30, 73.43] | 0.8127 | 0.0003 | [0.8121, 0.8133] | 0.7449 | 0.0027 | [0.7397, 0.7480] | 0.7110 | 0.0053 | [0.7047, 0.7206] | 0.7275 | 0.0015 | [0.7257, 0.7303] |
| AlphaFold2 + ProtVec | 1e-5 | 67.82 | 0.0305 | [67.76, 67.87] | 0.7491 | 0.0003 | [0.7485, 0.7497] | 0.6657 | 0.0013 | [0.6642, 0.6683] | 0.7158 | 0.0038 | [0.7080, 0.7195] | 0.6898 | 0.0012 | [0.6874, 0.6911] |
| Bio + ESM-2 | 1e-4 | 73.89 | 0.0309 | [73.84, 73.95] | 0.8186 | 0.0003 | [0.8181, 0.8193] | 0.7502 | 0.0023 | [0.7477, 0.7556] | 0.7164 | 0.0043 | [0.7063, 0.7207] | 0.7329 | 0.0012 | [0.7301, 0.7343] |
| Bio + ProtVec | 1e-4 | 67.04 | 0.0253 | [66.99, 67.08] | 0.7441 | 0.0003 | [0.7436, 0.7446] | 0.6649 | 0.0030 | [0.6593, 0.6681] | 0.6871 | 0.0092 | [0.6778, 0.7050] | 0.6758 | 0.0029 | [0.6727, 0.6814] |
| ESM-2 + ProtVec | 1e-4 | 73.52 | 0.0295 | [73.46, 73.57] | 0.8139 | 0.0003 | [0.8134, 0.8146] | 0.7498 | 0.0022 | [0.7465, 0.7544] | 0.7060 | 0.0042 | [0.6970, 0.7118] | 0.7272 | 0.0012 | [0.7245, 0.7289] |
| AlphaFold2 + Bio + ESM-2 | 1e-4 | 73.99 | 0.0317 | [73.91, 74.04] | 0.8204 | 0.0003 | [0.8198, 0.8210] | 0.7544 | 0.0033 | [0.7502, 0.7606] | 0.7112 | 0.0060 | [0.7004, 0.7197] | 0.7322 | 0.0017 | [0.7291, 0.7347] |
| AlphaFold2 + Bio + ProtVec | 1e-4 | 72.40 | 0.0290 | [72.35, 72.46] | 0.8027 | 0.0003 | [0.8021, 0.8033] | 0.7335 | 0.0025 | [0.7289, 0.7396] | 0.7038 | 0.0052 | [0.6906, 0.7143] | 0.7183 | 0.0016 | [0.7142, 0.7216] |
| AlphaFold2 + ESM-2 + ProtVec | 1e-4 | 73.28 | 0.0299 | [73.22, 73.34] | 0.8119 | 0.0003 | [0.8113, 0.8125] | 0.7439 | 0.0020 | [0.7402, 0.7475] | 0.7101 | 0.0039 | [0.7034, 0.7172] | 0.7266 | 0.0011 | [0.7247, 0.7287] |
| Bio + ESM-2 + ProtVec | 1e-4 | **74.18** | 0.0304 | [74.12, 74.25] | **0.8208** | 0.0003 | [0.8203, 0.8215] | **0.7557** | 0.0017 | [0.7523, 0.7588] | **0.7148** | 0.0032 | [0.7092, 0.7214] | **0.7347** | 0.0009 | [0.7330, 0.7366] |
| AlphaFold2 + ProtVec + Bio + ESM-2 | 1e-4 | 74.09 | 0.0298 | [74.03, 74.15] | 0.8207 | 0.0003 | [0.8201, 0.8213] | 0.7550 | 0.0026 | [0.7515, 0.7601] | 0.7133 | 0.0048 | [0.7040, 0.7190] | 0.7336 | 0.0013 | [0.7308, 0.7351] |

**Supplementary Table 18.** Robustness performance of the **GIN** model with pairwise, triple, and full embedding combinations using bootstrapping (100 iterations). For each configuration, results correspond to the best-tuned learning rate selected via the same testset.

| **Embedding** | **LR** | **Acc** | **Acc Std** | **Acc CI** | **AUC** | **AUC Std** | **AUC CI** | **Prec** | **Prec Std** | **Prec CI** | **Recall** | **Recall Std** | **Recall CI** | **F1** | **F1 Std** | **F1 CI** |
|---|---|---|---|---|---|---|---|---|---|---|---|---|---|---|---|---|
| AlphaFold2 + Bio | 1e-4 | 65.96 | 0.0259 | [65.92, 66.02] | 0.7294 | 0.0002 | [0.7290, 0.7299] | 0.6626 | 0.0028 | [0.6598, 0.6687] | 0.6505 | 0.0083 | [0.6333, 0.6591] | 0.6565 | 0.0029 | [0.6503, 0.6595] |
| AlphaFold2 + ESM-2 | 1e-4 | 65.82 | 0.0264 | [65.77, 65.88] | 0.7301 | 0.0002 | [0.7297, 0.7306] | 0.6457 | 0.0010 | [0.6437, 0.6471] | 0.7009 | 0.0035 | [0.6961, 0.7079] | 0.6722 | 0.0011 | [0.6705, 0.6745] |
| AlphaFold2 + ProtVec | 1e-4 | 63.04 | 0.0276 | [62.98, 63.09] | 0.6898 | 0.0002 | [0.6893, 0.6901] | 0.6275 | 0.0021 | [0.6230, 0.6307] | 0.6418 | 0.0086 | [0.6293, 0.6601] | 0.6345 | 0.0031 | [0.6299, 0.6411] |
| Bio + ESM-2 | 1e-4 | 66.46 | 0.0272 | [66.41, 66.51] | 0.7323 | 0.0003 | [0.7318, 0.7327] | 0.6636 | 0.0018 | [0.6590, 0.6663] | 0.6677 | 0.0057 | [0.6602, 0.6817] | 0.6656 | 0.0019 | [0.6629, 0.6702] |
| Bio + ProtVec | 1e-4 | 67.04 | 0.0253 | [66.99, 67.08] | **0.7441** | 0.0003 | [0.7436, 0.7446] | 0.6649 | 0.0030 | [0.6593, 0.6681] | 0.6871 | 0.0092 | [0.6778, 0.7050] | 0.6758 | 0.0029 | [0.6727, 0.6814] |
| ESM-2 + ProtVec | 1e-4 | 64.69 | 0.0265 | [64.63, 64.74] | 0.7159 | 0.0002 | [0.7154, 0.7163] | 0.6513 | 0.0026 | [0.6457, 0.6560] | 0.6323 | 0.0084 | [0.6175, 0.6497] | 0.6416 | 0.0031 | [0.6362, 0.6479] |
| AlphaFold2 + Bio + ESM-2 | 1e-5 | **67.13** | 0.0273 | [67.08, 67.18] | 0.7358 | 0.0003 | [0.7353, 0.7364] | 0.6574 | 0.0017 | [0.6555, 0.6612] | **0.7156** | 0.0057 | [0.7025, 0.7224] | **0.6852** | 0.0018 | [0.6812, 0.6874] |
| AlphaFold2 + Bio + ProtVec | 1e-4 | 66.75 | 0.0285 | [66.69, 66.79] | 0.7261 | 0.0003 | [0.7255, 0.7267] | **0.6745** | 0.0028 | [0.6688, 0.6783] | 0.6475 | 0.0076 | [0.6366, 0.6627] | 0.6607 | 0.0026 | [0.6566, 0.6658] |
| AlphaFold2 + ESM-2 + ProtVec | 1e-4 | 65.91 | 0.0262 | [65.86, 65.96] | 0.7294 | 0.0002 | [0.7290, 0.7299] | 0.6571 | 0.0011 | [0.6538, 0.6587] | 0.6657 | 0.0035 | [0.6601, 0.6777] | 0.6613 | 0.0012 | [0.6594, 0.6654] |
| Bio + ESM-2 + ProtVec | 1e-4 | 65.64 | 0.0278 | [65.59, 65.69] | 0.7205 | 0.0003 | [0.7201, 0.7210] | 0.6458 | 0.0027 | [0.6396, 0.6498] | 0.6928 | 0.0100 | [0.6786, 0.7157] | 0.6684 | 0.0032 | [0.6639, 0.6756] |
| AlphaFold2 + ProtVec + Bio + ESM-2 | 1e-3 | 58.68 | 0.0252 | [58.63, 58.73] | 0.5448 | 0.0003 | [0.5442, 0.5454] | 0.6747 | 0.0033 | [0.6706, 0.6830] | 0.3352 | 0.0047 | [0.3239, 0.3405] | 0.4479 | 0.0035 | [0.4393, 0.4520] |

Supplementary Tables 19 and 20 summarize the best-performing multimodal embedding combinations and their relative gains over unimodal baselines across all GNN models. Observing the different embedding combination results, we find that while strong single embeddings such as BioEmbedding already perform well for GraphSAGE, combining embeddings yields consistent and often substantial gains for GCN, GTN, and GIN, highlighting the complementary nature of structural, sequence-based, and language-derived protein representations. We observed GCN, GTN, and GIN show the largest consistent gains (for F1, Accuracy, and AUC), whereas SAGE is already near saturation with the best single embedding (BioEmbedding), so combinations provide minimal improvement. In contrast, other GNN models consistently benefit from carefully selected embedding combinations. Notably, full concatenation does not always provide optimal results, indicating that selective multimodal concatenation is more effective than indiscriminate feature aggregation.

**Supplementary Table 19.** Best-performing multimodal embedding combination per GNN model for PPI prediction, selected based on AUROC (primary metric) with F1-score and Accuracy as a tie-breaker.

| **Model** | **Best Concatenation** | **Accuracy (%)** | **AUROC** | **F1-score** |
|---|---|---|---|---|
| SAGE | BioEmbedding + ESM-2 | 79.29 | 0.8751 | 0.7893 |
| GAT | AlphaFold2 + BioEmbedding + ESM-2 | 75.56 | 0.8388 | 0.7564 |
| GCN | BioEmbedding + ESM-2 | 67.74 | 0.7468 | 0.6895 |
| GTN | BioEmbedding + ESM-2 + ProtVec | 74.18 | 0.8208 | 0.7347 |
| GIN | AlphaFold2 + BioEmbedding + ESM-2 | 67.13 | 0.7358 | 0.6852 |

**Supplementary Table 20.** Multimodal gain over the best single-embedding baseline for PPI prediction using 100-iteration bootstrap robustness. For each GNN model and evaluation metric, we report the best-performing single embedding, the best-performing multimodal (concatenated) embedding, and the absolute performance gain ($\Delta$ = Multi − Single). The absolute gain is computed as $\Delta$ = (performance of the best multimodal combination) − (performance of the best single embedding) under the same model architecture. Positive $\Delta$ values indicate that combining multiple embeddings improves performance relative to the strongest individual embedding, while values close to zero suggest that the model is already saturated with a single embedding.

| Model | Metric | Best Single Embedding | Best Multimodal Embedding | Performance Gain($\Delta$) |
|---|---|---|---|---|
| **Accuracy (%)** | | | | |
| SAGE | Accuracy | Bio (79.10) | Bio + ESM-2 (79.29) | +0.19% |
| GAT | Accuracy | Bio (75.58) | AlphaFold2 + Bio + ESM-2 (75.56) | −0.02% |
| GCN | Accuracy | Bio (66.47) | Bio + ESM-2 (67.74) | +1.27% |
| GTN | Accuracy | Bio (71.58) | Bio + ESM-2 + ProtVec (74.18) | +2.60% |
| GIN | Accuracy | ESM-2 (64.94) | AlphaFold2 + Bio + ESM-2 (67.13) | +2.19% |
| **AUROC** | | | | |
| SAGE | AUROC | Bio (0.8735) | Bio + ESM-2 (0.8751) | +0.16% |
| GAT | AUROC | Bio (0.8387) | AlphaFold2 + Bio + ESM-2 (0.8388) | +0.01% |
| GCN | AUROC | Bio (0.7312) | Bio + ESM-2 (0.7468) | +1.56% |
| GTN | AUROC | Bio (0.7935) | Bio + ESM-2 + ProtVec (0.8208) | +2.73% |
| GIN | AUROC | ESM-2 (0.7175) | Bio + ProtVec (0.7441) | +2.66% |
| **Precision** | | | | |
| SAGE | Precision | Bio (0.7956) | Bio + ESM-2 (0.8034) | +0.78% |
| GAT | Precision | Bio (0.7578) | AlphaFold2 + Bio + ESM-2 (0.7539) | −0.39% |
| GCN | Precision | Bio (0.6487) | Bio + ESM-2 (0.6646) | +1.59% |
| GTN | Precision | Bio (0.7192) | Bio + ESM-2 + ProtVec (0.7557) | +3.65% |
| GIN | Precision | ESM-2 (0.6440) | AlphaFold2 + Bio + ESM-2 (0.6574) | +1.34% |
| **Recall** | | | | |
| SAGE | Recall | Bio (0.7832) | Bio + ESM-2 (0.7756) | −0.76% |
| GAT | Recall | Bio (0.7520) | AlphaFold2 + Bio + ESM-2 (0.7590) | +0.70% |
| GCN | Recall | Bio (0.7186) | Bio + ESM-2 (0.7162) | −0.24% |
| GTN | Recall | Bio (0.7081) | Bio + ESM-2 + ProtVec (0.7148) | +0.67% |
| GIN | Recall | ESM-2 (0.6681) | AlphaFold2 + Bio + ESM-2 (0.7156) | +4.75% |
| **F1-score** | | | | |
| SAGE | F1 | Bio (0.7894) | Bio + ESM-2 (0.7893) | −0.01% |
| GAT | F1 | Bio (0.7549) | AlphaFold2 + Bio + ESM-2 (0.7564) | +0.15% |
| GCN | F1 | Bio (0.6819) | Bio + ESM-2 (0.6895) | +0.76% |
| GTN | F1 | Bio (0.7136) | Bio + ESM-2 + ProtVec (0.7347) | +2.11% |
| GIN | F1 | ESM-2 (0.6558) | AlphaFold2 + Bio + ESM-2 (0.6852) | +2.94% |
