# Supplementary material for: A multimodal human protein embeddings database: DeepDrug Protein Embeddings Bank (DPEB)
Source: NAR Genom Bioinform. 2026 Apr 28;8(2):lqag042. doi: 10.1093/nargab/lqag042 (PMC13137052; doi:10.1093/nargab/lqag042)
Supplement: lqag042_Supplemental_Files [file lqag042_supplemental_files.zip › DPEB-main.zip!/DPEB-main/references/References.docx]

DPEB	13

# References

1. Gregorio Alanis-Lobato, Miguel A Andrade-Navarro, and Martin H Schaefer. Hippie v2. 0: enhancing meaningfulness and reliability of protein–protein interaction networks. *Nucleic acids research*, 45(D1):D408–D414, 2017.
2. Diego Alonso-L´opez, Miguel A Guti´errez, Kiran P Lopes, Carlos Prieto, Ramon Santamar´ıa, and Javier De Las Rivas. Apid interactomes: providing proteome-based interactomes with controlled quality for multiple species and derived networks. *Nucleic acids research*, 44(W1):W529–W535, 2016.
3. Ehsaneddin Asgari and Mohammad R. K. Mofrad. Continuous distributed representation of biological sequences for deep proteomics and genomics. *PLOS ONE*, 10(11):1–15, 11 2015.
4. Andrius Bernatavicius, Martin S´ıcho, Antonius P. A. Janssen, Alan Kai Hassen, Mike Preuss, and Gerard

J. P. van Westen. Alphafold meets de novo drug design: Leveraging structural protein information in multitarget molecular generative models.	*Journal of Chemical Information and Modeling*, 64(21):8113–8122, 2024.

5. Muhao Chen, Chelsea J T Ju, Guangyu Zhou, Xuelu Chen, Tianran Zhang, Kai-Wei Chang, Carlo Zaniolo, and Wei Wang. Multifaceted protein–protein interaction prediction based on siamese residual rcnn. *Bioinformatics*, 35(14):i305–i314, 07 2019.
6. Muhao Chen, Chelsea J T Ju, Guangyu Zhou, Xuelu Chen, Tianran Zhang, Kai-Wei Chang, Carlo Zaniolo, and Wei Wang. Multifaceted protein–protein interaction prediction based on siamese residual rcnn. *Bioinformatics*, 35(14):i305–i314, July 2019.
7. The UniProt Consortium.	Uniprot: the universal protein knowledgebase in 2021. *Nucleic Acids Research*, 49(D1):D480–D489, 11 2020.
8. Christian Dallago, Konstantin Schu¨tze, Michael Heinzinger, Tobias Olenyi, Maria Littmann, Amy X Lu, Kevin K Yang, Seonwoo Min, Sungroh Yoon, James T Morton, and Burkhard Rost. Learned embeddings from deep learning to visualize and predict protein sets. *Current Protocols*, 1(5):e113, May 2021.
9. Das, S. and Chakrabarti, S. Classification and prediction of protein–protein interaction interface using machine learning algorithm. *Scientific Reports*, 11(1761), 2021.
10. Kevin Drew, John B Wallingford, and Edward M Marcotte.	hu.map 2.0: integration of over 15,000 proteomic experiments builds a global compendium of human multiprotein assemblies.	*Molecular Systems Biology*, 17:e10016, 2021.
11. Ahmed Elnaggar, Michael Heinzinger, Christian Dallago, Ghalia Rihawi, Yu Wang, Llion Jones, Tom Gibbs, Tamas

Feher, Christoph Angerer, Martin Steinegger, Debsindhu Bhowmik, and Burkhard Rost. Prottrans: Towards cracking the language of life’s code through self-supervised deep learning and high performance computing, 2021.

12. Jiahui Guan, Lantian Yao, Peilin Xie, Zhihao Zhao, Dian Meng, Tzong-Yi Lee, Junwen Wang, and Ying-Chih Chiang. Graph-rpi: predicting rna–protein interactions via graph autoencoder and self-supervised learning strategies. *Brief Bioinform*, 26(3):bbaf292, 2025.
13. Yanzhi Guo, Lezheng Yu, Zhining Wen, and Menglong Li. Using support vector machine combined with auto covariance to predict protein–protein interactions from protein sequences. *Nucleic Acids Research*, 36(9):3025– 3030, 04 2008.
14. Fan Hu, Yishen Hu, Weihong Zhang, Huazhen Huang, Yi Pan, and Peng Yin.	A multimodal protein representation framework for quantifying transferability across biochemical downstream tasks. *Advanced Science*,

p. 2301223, 2023.

15. Edward L Huttlin, Raphael J Bruckner, Joao A Paulo, Joe R Cannon, Lily Ting, Kristin Baltier, Greg Colby, Fana Gebreab, Melanie P Gygi, Hannah Parzen, et al. Architecture of the human interactome defines protein communities and disease networks. *Nature*, 545(7655):505– 509, 2017.
16. Edward L Huttlin, Lily Ting, Raphael J Bruckner, Fana Gebreab, Melanie P Gygi, John Szpyt, Stanley Tam, Gabriela Zarraga, Greg Colby, Kristin Baltier, et al. The bioplex network: a systematic exploration of the human interactome. *Cell*, 162(2):425–440, 2015.
17. K. Jha, S. Saha, and H. Singh. Prediction of protein–protein interaction using graph neural networks. *Sci Rep*, 12:8360, 2022.
18. John Jumper, Richard Evans, Alexander Pritzel, Tim Green, Michael Figurnov, Olaf Ronneberger, Kathryn Tunyasuvunakool, Russ Bates, Augustin Zidek, Anna Potapenko, et al. Highly accurate protein structure prediction with alphafold. *Nature*, 596(7873):583–589, 2021.
19. Sebesty´en Kamp, Giovanni Stracquadanio, and T. Ian Simpson. GNN-Suite: A graph neural network bench- marking framework for biomedical informatics. *arXiv preprint arXiv:2505.10711*, 2025. Comparative framework includes GCN, GAT, GraphSAGE, GIN, GTN, etc. for PPI networks:contentReference[oaicite:12]index=12.
20. T. S. Keshava Prasad, Renu Goel, Kumaran Kandasamy, Shivakumar Keerthikumar, Sameer Kumar, Suresh Mathivanan, Deepthi Telikicherla, Rajesh Raju, Beema Shafreen, Abhilash Venugopal, et al. Human protein reference database—2009 update. *Nucleic acids research*, 37(suppl 1):D767–D772, 2009.
21. Max Kotlyar, Chiara Pastrello, Zara Malik, and Igor Jurisica. Iid 2018 update: context-specific physical protein–protein interactions in human, model organisms and domesticated species.	*Nucleic acids research*, 47(D1):D581–D589, 2019.
22. Hang Li, Xiu-Jun Gong, Hua Yu, and Chang Zhou. Deep neural network based predictions of protein interactions using primary sequences. *Molecules*, 23(8):1923, 2018.
23. Zeming Lin, Halil Akin, Roshan Rao, Brian Hie, Zhongkai Zhu, Wenting Lu, Nikita Smetanin, Robert Verkuil, Ori Kabeli, Yaniv Shmueli, Allan dos Santos Costa, Maryam Fazel-Zarandi, Tom Sercu, Salvatore Candido, and

14	Sajol et al.

Alexander Rives. Evolutionary-scale prediction of atomic- level protein structure with a language model. *Science*, 379(6637):1123–1130, 2023.

24. Guofeng Lv, Zhiqiang Hu, Yanguang Bi, and Shaoting Zhang. Learning unknown from correlations: Graph neural network for inter-novel-protein interaction prediction. *CoRR*, abs/2105.06709, 2021.
25. Jiani Ma, Jiangning Song, Neil D. Young, Bill C. H. Chang, Pasi K. Korhonen, Tulio L. Campos, Hui Liu, and Robin B. Gasser. ’Bingo’: a large language model- and graph neural network-based workflow for predicting essential genes from protein data. *Briefings in Bioinformatics*, 25(1):bbad472, 2024. Compares GCN, GAT, GraphSAGE, GIN on PPI data:contentReference[oaicite:13]index=13.
26. Tom´as Mikolov, Ilya Sutskever, Kai Chen, Gregory S. Corrado, and Jeffrey Dean. Distributed representations of words and phrases and their compositionality. In Christopher J. C. Burges, L´eon Bottou, Zoubin Ghahramani, and Kilian Q. Weinberger, editors, *Advances in Neural Information Processing Systems 26: 27th Annual Conference on Neural Information Processing Systems 2013. Proceedings of a meeting held December 5-8, 2013, Lake Tahoe, Nevada, United States*, pp. 3111–3119, 2013.
27. Don Neumann, Soumyadip Roy, Fayyaz Ul Amir Afsar Minhas, and Asa Ben-Hur.	On the choice of negative examples for prediction of host-pathogen protein interactions. *Frontiers in Bioinformatics*, 2, December 2022.
28. Sandra Orchard, Mais Ammari, Bruno Aranda, Lionel Breuza, Leonardo Briganti, Fiona Broackes-Carter, Nancy H Campbell, Gayatri Chavali, Carol Chen, Noemi del Toro, Margaret Duesbury, Marine Dumousseau, Eugenia Galeota, Ursula Hinz, Marta Iannuccelli, Sruthi Jagannathan, Rafael Jimenez, Jyoti Khadake, Astrid Lagreid, Luana Licata, Ruth C Lovering, Birgit Meldal, Anna N Melidoni, Mila Milagros, Daniele Peluso, Livia Perfetto, Pablo Porras, Arathi Raghunath, Sylvie Ricard- Blum, Bernd Roechert, Andre Stutz, Michael Tognolli, Kim van Roey, Gianni Cesareni, and Henning Hermjakob. The mintact project–intact as a common curation platform for 11 molecular interaction databases. *Nucleic acids research*, 42(Database issue):D358—63, January 2014.
29. Lukasz Salwinski, Christopher S Miller, Adam J Smith, Frank K Pettit, James U Bowie, and David Eisenberg. The database of interacting proteins: 2004 update. *Nucleic acids research*, 32(suppl 1):D449–D451, 2004.
30. Bosheng Song, Xiaoyan Luo, Xiaoli Luo, Yuansheng Liu, Zhangming Niu, and Xiangxiang Zeng. Learning spatial structures of proteins improves protein–protein interaction prediction. *Briefings in Bioinformatics*, 23(2):bbab558, 01 2022.
31. Chris Stark, Bobby-Joe Breitkreutz, Teresa Reguly, Lorrie Boucher, Ashton Breitkreutz, and Mike Tyers. Biogrid: a general repository for interaction datasets. *Nucleic acids research*, 34(suppl 1):D535–D539, 2006.
32. Martin Steinegger and Johannes S¨oding. Clustering huge protein sequence sets in linear time. *Nature Communications*, 9(1):2542, 2018.
33. Damian Szklarczyk, Rebecca Kirsch, Mikaela Koutrouli, Katerina Nastou, Farrokh Mehryary, Radja Hachilif,

Annika L Gable, Tao Fang, Nadezhda T Doncheva, Sampo Pyysalo, Peer Bork, Lars J Jensen, and Christian von Mering. The string database in 2023: protein-protein association networks and functional enrichment analyses for any sequenced genome of interest. *Nucleic Acids Research*, 51(D1):D638–D646, Jan 2023.

34. Jiahui Wu, Bo Liu, Jidong Zhang, Zhihan Wang, and Jianqiang Li. Dl-ppi: a method on prediction of sequenced protein–protein interaction based on deep learning. *BMC Bioinformatics*, 24(1):473, 2023.
35. Jiahui Wu, Bo Liu, Jidong Zhang, Zhihan Wang, and Jianqiang Li. Dl-ppi: a method on prediction of sequenced protein–protein interaction based on deep learning. *BMC Bioinformatics*, 24(1), December 2023.
36. Mingda Xu, Peisheng Qian, Ziyuan Zhao, Zeng Zeng, Jianguo Chen, Weide Liu, and Xulei Yang. Graph neural networks for protein-protein interactions – a short survey. *arXiv preprint arXiv:2404.10450*, 2024. Surveys GNN-based PPI methods; highlights GCN- based (e.g. GraphSAGE, GIN) and GAT-based model families:contentReference[oaicite:14]index=14.
37. Yang Lei, Xia Jun-Feng, and Gui Jie. Prediction of protein-protein interactions from protein sequence using local descriptors. *Protein & Peptide Letters*, 17(9), 2010.
38. You, ZH., Zhu, L., Zheng, CH., and et al. Prediction of protein-protein interactions from amino acid sequences using a novel multi-scale continuous and discontinuous feature set. *BMC Bioinformatics*, 15 (Suppl 15)(S9), 2014.
39. Bin Yu, Cheng Chen, Xiaolin Wang, Zhaomin Yu, Anjun Ma, and Bingqiang Liu. Prediction of protein–protein interactions based on elastic net and deep forest. *Expert Systems with Applications*, 176:114876, 2021.
40. Xin Zeng, Fan-Fang Meng, Meng-Liang Wen, Shu-Juan Li, and Yi Li. Gnngl-ppi: multi-category prediction of protein- protein interactions using graph neural networks based on global graphs and local subgraphs. *BMC Genomics*, 25(1):406, 2024.
41. Xin Zeng, Fan-Fang Meng, Meng-Liang Wen, Shu-Juan Li, and Yi Li. Gnngl-ppi: multi-category prediction of protein- protein interactions using graph neural networks based on global graphs and local subgraphs. *BMC Genomics*, 25(1), May 2024.
42. Hang Zhou, Weikun Wang, Jiayun Jin, Zengwei Zheng, and Binbin Zhou. Graph neural network for protein– protein interaction prediction: A comparative study. *Molecules*, 27(18):6135, 2022. Benchmarks GCN and GAT (among others) as widely-used GNN models for PPI prediction:contentReference[oaicite:15]index=15.
43. Yu Zhen Zhou, Yun Gao, and Ying Ying Zheng. Prediction of protein-protein interactions using local description of amino acid sequence. In Mark Zhou and Honghua Tan, editors, *Advances in Computer Science and Education Applications*, pp. 254–262, Berlin, Heidelberg, 2011. Springer Berlin Heidelberg.
44. Zhu-Hong You, Xiao Li, and Keith CC Chan. An improved sequence-based prediction protocol for protein- protein interactions using amino acids substitution matrix and rotation forest ensemble classifiers. *Neurocomputing*, 228:277–282, 2017. Advanced Intelligent Computing: Theory and Applications.
